# 2D Material light–emitting transistor with a dynamically controllable emission location for optimized waveguide coupling on silicon

**DOI:** 10.1126/sciadv.aeb8783

**Published:** 2026-04-03

**Authors:** Chen Li, Yongzhuo Li, Yutong Zhong, Yuqian Tang, Cun-Zheng Ning

**Affiliations:** ^1^ Department of Electronic Engineering, Tsinghua University, 100084 Beijing, China.; ^2^ College of Integrated Circuits and Optoelectronic Chips, Shenzhen Technology University, 518118 Shenzhen, Guangdong, China.; ^3^ Frontier Science Center for Quantum Information, 100084 Beijing, China.; ^4^ Beijing National Research Center for Information Science and Technology, 100084 Beijing, China.; ^5^ Tsinghua International Center for Nano-Optoelectronics, Tsinghua University, 100084 Beijing, China.

## Abstract

Two-dimensional semiconductors are promising for silicon-compatible on-chip light sources. However, substantial challenges persist regarding component performance, functional integration, and the lack of optimal system architectures. Specifically, the precise integration of nanoscale light sources with photonic components, such as waveguides, remains difficult because of stringent alignment requirements. Here, we demonstrate a silicon-compatible MoTe_2_ light-emitting transistor (LET) featuring both electrical switching and dynamically reconfigurable light emission. The device operates as an ambipolar transistor with a high on/off ratio (>10^5^) and uses a voltage-tunable dynamic p-i-n junction, enabling electrically programmable electroluminescence (EL) positioning across the 15-micrometer channel. We further integrate the LET with a silicon waveguide acting as a back gate. The emission around 1300 nanometers is dynamically tuned and coupled into the waveguide, collected via grating couplers. We achieve an electrically reconfigurable EL localization in silicon-integrated devices and a record efficiency of 67%. This work enables the development of reconfigurable photonic circuits by overcoming integration bottlenecks.

## Teaser

A dynamically reconfigurable light-emitting transistor overcomes integration bottlenecks in silicon photonics.

## INTRODUCTION

Transition metal dichalcogenides (TMDCs) have emerged as a promising platform for the next-generation optoelectronic integrated circuits due to their distinctive electronic and optical properties (*1*, *2*). Their exceptional excitonic properties, combined with atomic-scale thickness, offer substantial potential for the miniaturization of light sources in photonic integrated circuits. Moreover, the absence of dangling bonds on their surfaces enables seamless heterogeneous integration with arbitrary substrates via van der Waals interactions. Recent advances in combining TMDCs photoluminescence (PL) with photonic crystal cavities (*3*–*7*), waveguides (*8*–*10*), and microdisks (*11*–*13*) provide compelling evidence of their substantial potential for silicon-compatible light source engineering.

Nevertheless, the silicon-integrated electroluminescence (EL) of TMDCs remains underexplored, primarily because of current EL devices requiring complex electrical injection structures, including metallic gates (*14*–*16*) or ionic liquid gating (*17*, *18*) for carrier modulation, or relying on interdigitated electrodes for electric field–induced light emission (*19*). These device architectures are fundamentally incompatible with silicon photonic integration paradigms. Furthermore, the electroluminescent zones of devices are predominantly localized near electrode edges, resulting in poor coupling efficiency to silicon waveguides or optical cavities. In addition, the static nature of the emission position constitutes a critical bottleneck, as optical coupling efficiency is strictly limited by the precise spatial alignment between light-emitting sources and passive optical components. Once integrated, the coupling efficiency becomes fixed and cannot be postoptimized or dynamically adjusted to meet varying operational requirements. This limitation severely constrains device performance and restricts application flexibility, particularly for programmable photonic circuits requiring reconfigurability.

Here, we demonstrate a few-layer MoTe_2_-based light-emitting transistor (LET) integrated with dynamically tunable waveguides on silicon, seamlessly combining electrical switching and light emission capabilities into a single device. The recombination zone is tunable via gate voltage relative to the electrode edge, enabling the ~1300-nm emission to be efficiently coupled to the silicon waveguide. The proposed device features a back-gated transistor architecture with MoTe_2_ as the channel material. To avoid the Fermi pinning effect between the electrode and MoTe_2_, gold pads were transferred onto the MoTe_2_ as drain (D) and source (S) electrodes to form van der Waals contacts (*20*). The contact barriers at the interfaces are modulated by a back gate (G), enabling precise control over the injection ratio of electrons and holes. The measured on/off ratios of the MoTe_2_ LET can reach as high as 1.65 × 10^6^ and 5.56 × 10^5^ for n-type and p-type operation, respectively. Leveraging this ambipolar switching capability, we spatially manipulate the electron-hole recombination zone by tuning the effective potential distribution in the channel through gate and bias voltage. In this way, we realize the free movement of the EL spot along the entire channel length of 15 μm. Furthermore, we migrate the architecture of the MoTe_2_ LET to the silicon waveguide, achieving a dynamically tunable coupling that surpasses the capabilities of conventional heterogeneous integration light sources. Our results establish a previously unexplored route for next-generation near-infrared silicon-integrated optoelectronic devices with both electrical and optical outputs. This paradigm shift not only circumvents traditional integration bottlenecks but also opens avenues for electrically reconfigurable photonic circuits.

## RESULTS

### Device architecture and electrical properties

**Fig. 1.**
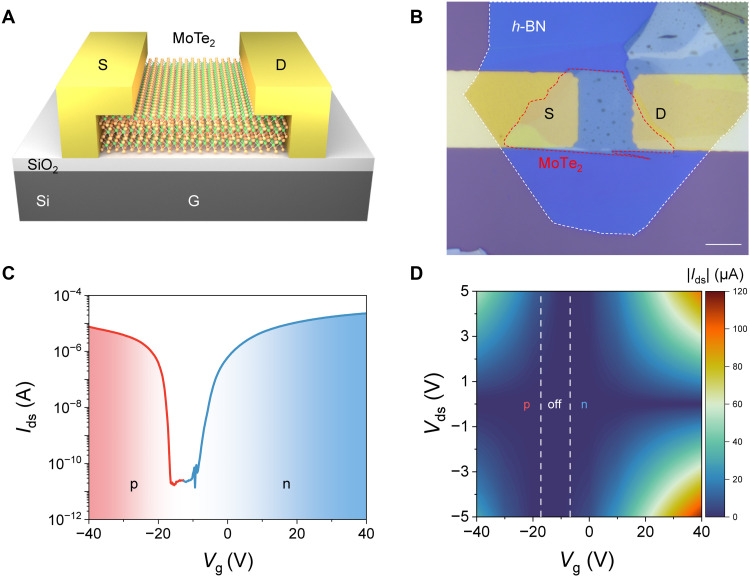
Structure and electrical characterizations of MoTe_2_ LETs. (**A**) Schematic of the MoTe_2_ LETs. (**B**) Optical microscope image of the device. Scale bar, 10 μm. (**C**) Transfer curve at *V*_ds_ = 1.0 V. (**D**) Contour plot of the *I*_ds_ versus gate voltage *V*_g_ and bias voltage *V*_ds._

Figure 1 (A and B) shows the schematic and optical image of the MoTe_2_ LET. The device consists of a few-layer MoTe_2_ on a silicon substrate (G) with a 300-nm-thick SiO_2_ layer as the gate oxide. The MoTe_2_ channel is contacted by two Au electrodes (D and S) separated by a distance of 15 μm and is encapsulated by a thin *h*-BN protective layer (not shown in Fig. 1A). MoTe_2_ is known to exhibit high mobilities ranging from 0.3 to 20 cm^2^ V^−1^ s^−1^ for holes and 0.03 to 30 cm^2^ V^−1^ s^−1^ for electrons (*21*, *22*), as well as a narrow bandgap of 0.93 to 1.1 eV as the thickness decreases from bulk to monolayer (*23*). The first function of MoTe_2_ LETs is ambipolar field-effect transistor (FET) characteristics, which means that both electron and hole doping can be achieved by different gate voltages. Figure 1C shows the electrical transfer curve (*I*_ds_–*V*_g_) of the MoTe_2_ LET with a bias voltage of *V*_ds_ = 1 V. The current curve of the MoTe_2_ LET exhibits a negative neutral point (off region), indicating that the few-layer MoTe_2_ is intrinsically n-doped, which is consistent with previous studies (*22*, *24*). *I*_ds_ varies from a minimum of 13.9 pA at *V*_g_ = −9.4 V to a maximum of 22.9 μA for electrons at *V*_g_ = 40 V and 7.73 μA for holes at *V*_g_ = −40 V. The measured on/off ratios can reach as high as 1.65 × 10^6^ and 5.56 × 10^5^ for the n-type and p-type regimes, respectively. The field-effect mobility can be calculated according to the following equation (*25*)

$$\mu =\frac{L}{W}\times \frac{h}{\varepsilon_{0}\varepsilon_{r}V_{\text{ds}}}\times \frac{dI_{\text{ds}}}{dV_{g}}$$ (1)

where *L* (15 μm) and *W* (20 μm) are the MoTe_2_ channel length and width, *h* (300 nm) and ε_r_ (3.9) are the thickness and dielectric constant of SiO_2_, and d*I*_ds_/d*V*_g_ is the slope of the transfer curve in the linear region. When *V*_ds_ = 1 V, the electron (hole) mobility is extracted as 39.1 (23.2) cm^2^ V^−1^ s^−1^, which surpasses most ambipolar MoTe_2_ FETs (*24*, *26*–*28*). The threshold voltages of the electron (*V*_th,n_) and hole (*V*_th,p_) are −6.8 V and −17.2 V, which can be extracted based on the second derivative logarithmic method (*29*). Figure 1D presents a contour map of *I*_ds_ as a function of *V*_ds_ and *V*_g_, while the output curves (*I*_ds_–*V*_ds_) are shown in fig. S1. The output curves under the hole doping regime display nonlinear and asymmetric behavior, a signature of the Schottky contacts between electrodes and MoTe_2_, which is directly visualized in the photocurrent mapping shown in fig. S2. In contrast, the electron doping regime yields linear output characteristics due to the lowered Schottky barrier with the high doping levels. The current of n-doped (*V*_g_ = 40 V) is higher than that of p-doped (*V*_g_ = −40 V) with the same bias voltage, which indicates that the Fermi level aligns closer to the conduction band of MoTe_2_. Overall, these results demonstrate that both carrier concentration and Schottky barrier can be effectively modulated by the gate voltages. This performance is facilitated by the transferred Au electrode technique, which mitigates the Fermi pinning effect commonly observed at metal-semiconductor interfaces.

### The principle of ambipolar injection

**Fig. 2.**
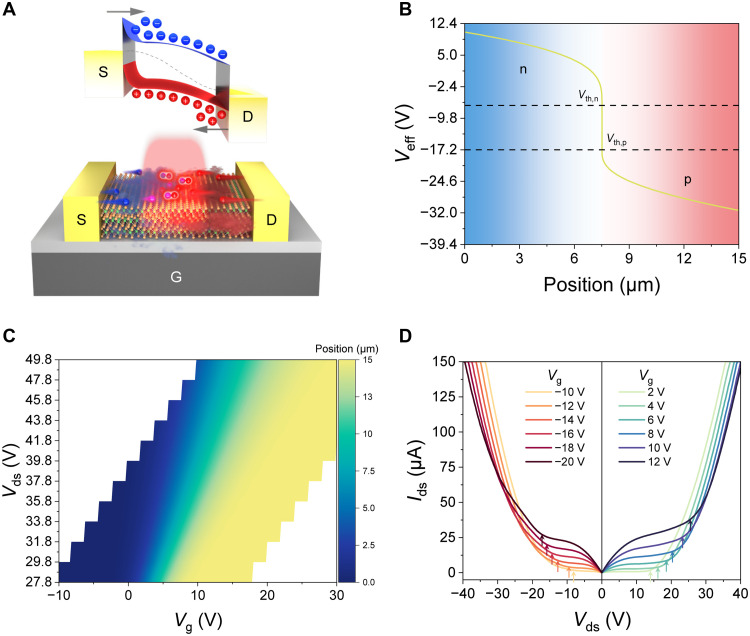
Principles and electrical characterizations of ambipolar injection. (**A**) Schematic diagram of band structure (top) and injection type (bottom) for ambipolar injection. The Fermi level of the entire channel is represented by the dashed line. (**B**) Distribution of *V*_eff_ under configuration to dynamic p-i-n junction at *V*_ds_ = 41.8 V, *V*_g_ = 10.4 V. (**C**) The distribution relationship of the position where *V*_eff_ = 0 V on a simultaneous change in *V*_ds_ and *V*_g_. (**D**) *I*_ds_-*V*_ds_ curves in p type (left) and n type (right) exhibit ambipolar characteristics. The arrows could be defined as the beginning of ambipolar transport.

In conventional FETs, and even in many reported ambipolar FETs, typically only one type of charge carrier dominates the channel at a given time. In contrast, our proposed LETs enable the simultaneous injection of both electrons and holes into the channel, controlled by a single G. Under low bias voltages, the single-gate configuration produces an approximately uniform distribution of carriers (electrons or holes). However, as the bias voltage increases, carrier distribution along the long MoTe_2_ channel becomes nonuniform. The local doping state within the MoTe_2_ channel is determined by the effective gate voltage of *V*_eff_ (*x*) = *V*_g_ – *V*_ch_ (*x*) according to the pinch-off effect, where *V*_ch_ (*x*) represents the electric potential of the channel at a lateral position *x*. When *V*_ds_ increases to a point where its influence along the channel can no longer be neglected, the *V*_eff_ near the D and S electrodes becomes unequal. This disparity consequently leads to variations in carrier concentration or different types of carrier doping. Therefore, in our proposed MoTe_2_ LETs, a single gate can simultaneously inject both electrons and holes into the channel without the assistance of ionic liquid, thereby demonstrating its potential for EL. This capability overturns the traditional notion that double gates are required and showcases their compatibility with subsequent silicon-based processes. To simplify the analysis, we focus on the discussion about the case of *V*_ds_ > 0 V and *V*_g_ > *V*_th,n_, where electrons are injected into the channel from the S terminal as the primary charge carriers at low *V*_ds_. In addition, we assume that the D and S electrodes exhibit identical contact conditions with few-layer MoTe_2_. Carrier injection modes can be categorized into two types: unipolar (fig. S3) and ambipolar injection (Fig. 2A), which exhibit distinct EL characteristics. We analyze the distribution of *V*_eff_ (*x*) under various *V*_ds_ and *V*_g_ conditions to gain an in-depth understanding of EL phenomena and lateral carrier injection dynamics. We define the positions of S and D electrodes as *x* = 0 and *x* = *L*, respectively. The distribution of *V*_eff_ (*x*) can be extracted from the transfer curve (Fig. 1C) using the following equation (*30*)

$$x_{0}=L\cdot \int_{V_{g}-V_{s}}^{V_{\text{eff}}\left(x_{0}\right)}I_{\text{ds}}\left(V_{\text{eff}}\right)dV_{\text{eff}}/\int_{V_{g}-V_{d}}^{V_{g}-V_{s}}I_{\text{ds}}\left(V_{\text{eff}}\right)dV_{\text{eff}}$$ (2)

where *x*_0_ is the position of *V*_eff_ to be solved. When *V*_th,p_ + *V*_ds_ > *V*_g_ > *V*_th,n_, the effective gate voltage at the S terminal satisfies *V*_eff_ (0) = *V*_g_ > *V*_th,n_ (n type), while at the D terminal, *V*_eff_ (*L*) = *V*_g_ – *V*_ds_ < *V*_th,p_ (p type). In this case, electrons are injected from the S terminal and holes from the D terminal, leading to radiative recombination within the channel. Figure 2B illustrates the formation of a dynamic p-i-n junction along the channel. The distribution of *V*_eff_ (*x*) along the channel reveals three distinct areas: the electron concentration gradient decreasing region, the undoped region, and the hole concentration gradient increasing region. The blue and red regions in Fig. 2B represent n-type and p-type doping, respectively. Notably, the abrupt change of *V*_eff_ occurs between the threshold voltages of the electron and the hole. This area is regarded as the central region of the transition zone between the doped regions. The position of this transition zone can be well controlled by adjusting *V*_ds_ and *V*_g_ (Fig. 2C). When *V*_ds_ = 41.8 V and *V*_g_ = 10.4 V, the transition region appears in the middle of the channel. Specifically, the transition region moves from the S to the D terminal with decreasing *V*_ds_ or increasing *V*_g_ (fig. S3). Therefore, the EL position can be spatially manipulated by tuning the injection ratio of electrons and holes via *V*_ds_ and *V*_g_, allowing the emission zone to be kept away from the metallic electrodes to avoid optical absorption and quenching losses (*31*). The phenomenon of ambipolar injection can be observed through the output curves at high *V*_ds_. The presence of defect charges at the SiO_2_ and its interface strongly affects the characteristics of the intrinsic MoTe_2_ channel, such as the threshold voltage. We characterize the output curves in n type and p type with the transfer curve (fig. S4), as shown in Fig. 2D. The output curves display distinct linear and saturation regions at low *V*_ds_. However, as *V*_ds_ exceeds *V*_g_ – *V*_th_, *I*_ds_ exhibits a nonlinear growth beyond the saturation state. This is considered a feature of the accumulation saturation of majority carriers and the gradual injection of minority carriers, which has been corroborated in ambipolar FETs (*18*, *32*–*35*).

### Dynamically controllable position of EL

**Fig. 3.**
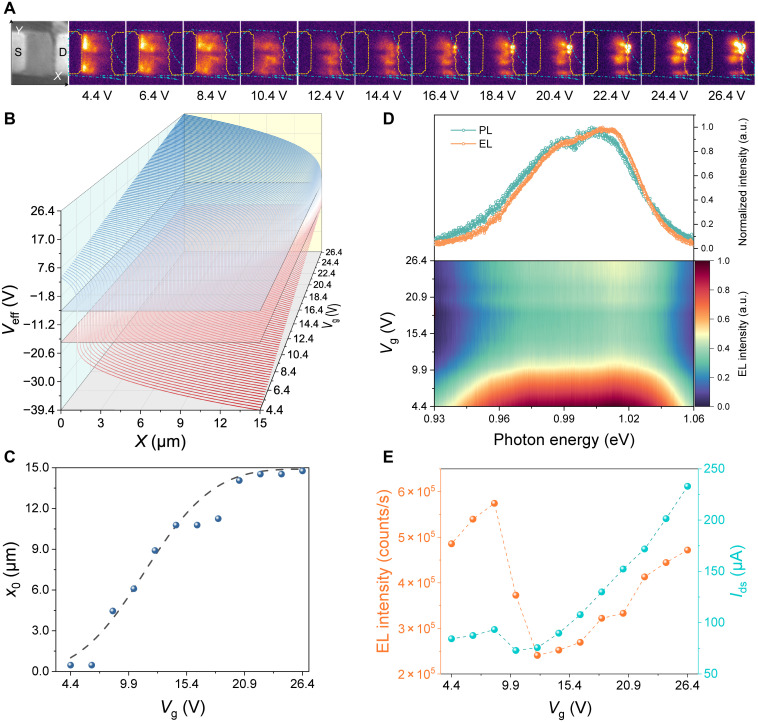
Electrical tunability of EL of MoTe_2_ LETs. (**A**) Charge-coupled device (CCD) images (false color) of device and EL emission at fixed *V*_ds_ = 43.8 V and different *V*_g_. The emission region moves from the S (left) to the D (right) with increasing *V*_g_. (**B**) Distribution of *V*_eff_ under different *V*_g_ with *V*_ds_ of 43.8 V in ambipolar injection regime. The blue (red) plane represents the equipotential surface of the *V*_th,n_ (*V*_th,p_). (**C**) EL positions along the channel at different *V*_g_ [The blue points are extracted from (A), and the dashed line is extracted from (B)]. (**D**) Normalized PL and EL spectra of the device (top). The PL spectrum was excited by a 633-nm laser. The EL spectrum was measured at *V*_ds_ = −46.2 V, *V*_g_ = −31 V. EL spectra with fixed *V*_ds_ = 43.8 V and different *V*_g_ (bottom). (**E**) EL intensity (orange) and *I*_ds_ (cyan) as a function of *V*_g_. a.u., arbitrary units.

In contrast to conventional electroluminescent devices, which have fixed luminescent regions without adjustability, our proposed LET enables dynamic control over the position of light emission, offering superior compatibility with silicon photonics. The EL image of the device serves as a more visual and intuitive representation, effectively indicating the region associated with radiative recombination. Therefore, the EL of MoTe_2_ LET offers crucial insights into the research on lateral carrier injection, transport mechanisms, and recombination processes. We use the thermoelectric-cooled InGaAs charge-coupled device camera to capture the EL images. Figure 3A (left) displays the image of MoTe_2_ LET illuminated by a mercury lamp. We define the direction from the S to the D terminal as the positive *x* axis, with the S edge at *x* = 0 and the D edge at *x* = *L* = 15 μm, while the direction perpendicular to the channel is defined as the *y* axis. Because of the ambipolar characteristic (Fig. 1C), when *V*_g_ gradually increases from 4.4 to 26.4 V with *V*_ds_ fixed at 43.8 V, *V*_eff_ near the S terminal follows the change of *V*_g_, exhibiting the n-type doping; the *V*_eff_ near the D terminal decreases from −39.4 to −17.4 V, exhibiting the p-type doping. The position of EL moves toward the D terminal with the increase of *V*_g_, as shown in Fig. 3A. In particular, when the current under n doped is equal to the current under p doped, that is, *I*_e_ = *I*_h_ = 5 μA (Fig. 1C, where *I*_e_ is the *I*_ds_ under *V*_g_ = 10.4 V and *I*_h_ is the *I*_ds_ under *V*_g_ = −33.4 V), the EL position is located in the center of the channel. In this balanced state, the luminescent area spans nearly the entire channel (~300 μm^2^), indicating that electrons injected from the S terminal and holes injected from the D terminal recombine efficiently across the device (Fig. 2A). Furthermore, reversing carrier injection polarity (fig. S5) maintains EL position tunability, confirming gate-defined bidirectional control. In contrast, unipolar injection (fig. S6) localizes EL near electrode edges due to limited minority carrier participation, highlighting the necessity of ambipolar injection for integration of silicon photonics.

To quantitatively resolve the spatial carrier distribution, we use Eq. 2 to calculate the *V*_eff_ across the channel. Under the fixed *V*_ds_ = 43.8 V, the distribution of *V*_eff_ along the channel with different *V*_g_ is shown in Fig. 3B. The results reveal an abrupt transition in *V*_eff_ at a specific position *x*_0_ within the channel. Consequently, electrons and holes accumulate on opposite sides of this point, forming a dynamic p-i-n junction separated by a narrow transition zone. Recombination occurs at this critical plane (*x* = *x*_0_), where the effective gate potential approaches zero after correcting for the threshold voltage (*36*). The position of *x*_0_ gradually moves from the S to the D terminal as *V*_g_ increases, which is consistent with the movement observed in the EL images. Experimentally, the EL position is defined as the location of peak intensity, obtained by integrating the EL data from Fig. 3A along the direction perpendicular to the channel (transverse axis). Figure 3C presents the dependence of the EL position on *V*_g_, while the dashed line shows the calculated position of the carrier density transition (*x*_0_). The consistency between the EL observations and electrical calculations confirms that the EL mechanism arises from the radiative recombination of ambipolarly injected carriers. Moreover, the EL images provide a direct visualization of the variations in the ratio of electron and hole injection, highlighting the dynamic behavior of charge carriers. We also evaluated the long-term operational stability of the MoTe_2_ LET. Continuous carrier injection was found to maintain the spatial stability of the EL emission. However, the encapsulation approach used here is less mature than that reported for ambient-sensitive black phosphorus–based light-emitting diode, which has achieved operational lifetime exceeding 15,000 hours (*37*). Therefore, the performance of continuous emission is substantially limited by thermal effects (fig. S8).

The top panel of Fig. 3D shows the normalized spectra of PL and EL with ambipolar injection, which indicates that light emission originates from radiative recombination rather than thermal radiation (fig. S9). The unique band structure of MoTe_2_ (*38*) enables further modulation of EL spectral features by bias and gate voltages. The bottom panel of Fig. 3D displays gate-tunable EL spectra corresponding to each *V*_g_ in Fig. 3A. Evidently, as *V*_g_ increases, the emission peak at 1.02 eV (K-K direct transition) is enhanced relative to the 0.97 eV peak (Λ-K indirect transition). The peak shift reflects that the transfer of the electron population from the Λ-valley to the K-valley under high doping levels, selectively amplifying the direct bandgap transition—a mechanism promising for wavelength-agile light sources. Figure 3E shows the relationship between EL intensity (the integrated background-subtracted EL intensity per second extracted from Fig. 3A) and injection current. The modulation of *V*_g_ disrupts the equilibrium of carrier injection, leading to an overinjection of either electrons or holes. The imbalance of carrier injection causes the emission to be localized near the electrode, which leads to the quenching of excitons. If the distance between the recombination region and the electrode is sufficiently small, excess carriers may escape into the electrode, which contributes to the drain current without generating light. It can be observed that when the EL region is centered in the channel (away from the electrodes), the EL intensity does not show a notable advantage. This is because the *I*_ds_ varies substantially under different *V*_g_. Specifically, at *V*_g_ = 4.4 V, a nonnegligible EL region remains within the channel. However, if *V*_g_ is further reduced, the EL region moves closer to the S terminal, leading to increased nonradiative losses and reduced EL efficiency. Therefore, balanced carrier injection maximizes the recombination zone’s distance from electrode contacts, suppressing loss of quenching and improving efficiency of EL by ~3× (estimated from Fig. 3E).

### Waveguide-integrated MoTe_2_ LET

**Fig. 4.**
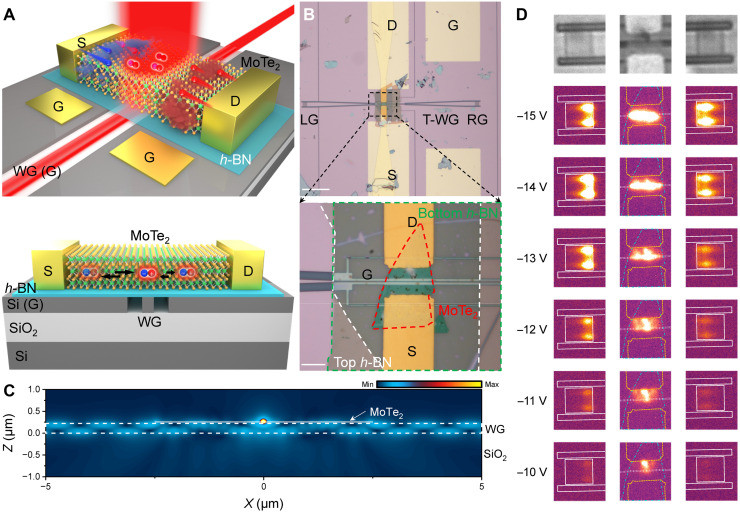
The waveguide-integrated MoTe_2_ LET. (**A**) Schematic of the waveguide-integrated MoTe_2_ LETs (top). Cross-sectional schematic of the device (bottom). (**B**) Optical image of the fabricated device. Top: Whole silicon structures with the straight waveguide, tapered waveguides, and grating couplers. Scale bar, 100 μm. Bottom: Zoomed-in region of the device. Scale bar, 10 μm. LG, left grating coupler; RG, right grating coupler; T-WG, tapered waveguide. (**C**) The simulation of the cross section of the electric field distribution. A dipole source along the *y*-axis direction is placed at the center of the MoTe_2_ layer. The *h*-BN (30 nm), MoTe_2_ (10 nm), and *h*-BN (30 nm) are placed on the silicon waveguide in sequence. (**D**) CCD images of the device, grating couplers, and EL emission of the center, left grating coupler, and right grating coupler at fixed *V*_g_ = −12 V and different *V*_ds_.

Owing to the dynamical tunability of the EL spatial profile through a single G, our proposed MoTe_2_ LET offers notable advantages for integration with a silicon-based waveguide. Here, we further demonstrate a waveguide-integrated MoTe_2_ LET, as shown in Fig. 4A. In this innovative design, a silicon waveguide serves a dual purpose, functioning simultaneously as an optical mode confiner and a G electrode. This architecture eliminates parasitic absorption associated with traditional metal gates while preserving effective electrostatic gating capabilities. By using photonic crystal grating couplers (*39*) and patterning electrical isolation slots, the silicon slab beneath the device and the waveguide itself are maintained at an equipotential when the *V*_g_ is applied to the G (Fig. 4B). Further details are provided in the fig. S10. Furthermore, to enhance carrier modulation, *h*-BN was used as a dielectric layer, leveraging its atomically smooth interface and superior capacitive properties. Consequently, we have in situ constructed an ambipolar transistor on the waveguide with a width of 520 nm, which acts as a fundamental building block for the development of on-chip waveguide-integrated electrical logic devices. The basic electrical characteristics are presented in fig. S11, and fabrication details are provided in Materials and Methods. Figure 4B displays optical microscope images of the fabricated device. The emission from the MoTe_2_ LET is directly coupled into the waveguide and propagates to grating couplers at both ends via a 100-μm straight waveguide and 250-μm tapered waveguides. The near-field coupling of emission from the MoTe_2_ into the waveguide was simulated using three-dimensional (3D) finite-difference time-domain methods, as shown in Fig. 4C. The MoTe_2_ LET was modeled as an electrical dipole oscillating along the *y*-axis direction (perpendicular to the waveguide). As predicted, the emission from the MoTe_2_ couples efficiently into the waveguide and propagates along the *x* axis. Figure 4D shows EL images captured from the LET (above the waveguide) and the grating couplers at both ends, under a fixed *V*_g_ = −12 V and different *V*_ds_. Notably, in this experiment, modulation of the emission position is achieved by adjusting the *V*_ds_ while maintaining a constant *V*_g_. Unlike the alternative approach of varying *V*_g_ at a fixed *V*_ds_—which primarily modifies carrier injection at the D terminal—this method allows the emission position to move gradually near the waveguide, facilitating systematic observation of coupling efficiency variations. The strong EL intensities from the grating couplers indicate that the EL generated by the device is effectively coupled into the waveguide. As *V*_ds_ decreases, the EL position moves toward the D terminal (away from the waveguide center), resulting in a gradual decrease in the output intensity from the grating couplers.

**Fig. 5.**
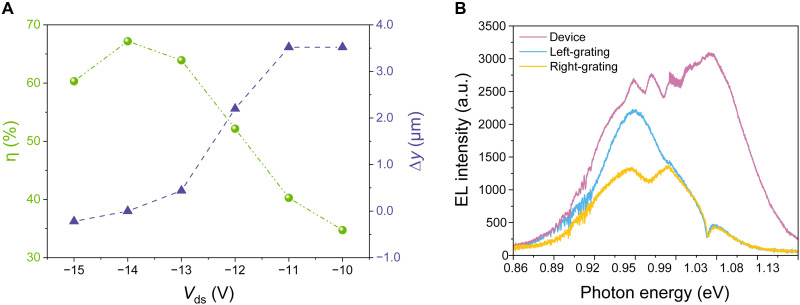
The EL controllable characteristics of the waveguide-integrated MoTe_2_ LET. (**A**) The fraction η of EL photons collected from the grating couplers and the distance from the EL position to the center of the waveguide at fixed *V*_g_ = −12 V and different *V*_ds_. (**B**) EL spectra collected from the device and the two output grating couplers at *V*_g_ = −12 V and *V*_ds_ = −14 V.

The variation of the output intensity from the grating couplers is mainly caused by the movement of the EL position rather than simply reducing the output intensity of the light source. We estimate the efficiency η of the waveguide-integrated MoTe_2_ LET and define it as $\eta =\left[I_{\text{LG}+\text{RG}}/\left(I_{\text{LG}+\text{RG}}+I_{\text{LET}}\right)\right]$, where *I*_LG + RG_ (*I*_LET_) denotes the integrated emission intensity over the entire area and wavelength collected from the two grating couplers (the MoTe_2_ LET), respectively (*40*). Notably, the photonic structure efficiency η is an aggregate metric that incorporates factors such as the coupling efficiency between emitter and waveguide, propagation loss, the coupling efficiency of the grating coupler, the extraction efficiency of the objective lens, etc. It reflects the performance from emission to final extraction, which represents a system-level efficiency rather than an intuitive coupling efficiency. As seen in section S3, the device achieves a maximum coupling efficiency of 21.2% at 1329 nm. The efficiency η could be adjusted from 35 to 67% at fixed *V*_g_ = −12 V and different *V*_ds_, as shown in Fig. 5A. To analyze this dependence, we define the offset ∆*y* as the deviation between the waveguide center and the maximum EL position. This peak position is determined by integrating the EL intensity along the waveguide direction, using data extracted from Fig. 4D. When the EL emission is localized at the center of the waveguide at *V*_ds_ = −14 V and *V*_g_ = −12 V, η reaches its maximum value of 67%. This record-high efficiency is attributed to the combination of dynamically tunable EL positioning and our innovative device architecture, which uses the silicon waveguide as a G. This design eliminates the absorption losses typically associated with additional graphene or metal gates, allowing the waveguide-integrated MoTe_2_ LET to substantially surpass the η of existing waveguide-integrated 2D-material EL devices (*40*–*46*). A detailed performance comparison is provided in table S2. Figure 5B plots the EL spectra collected from the MoTe_2_ LET and the grating couplers at *V*_ds_ = −14 V and *V*_g_ = −12 V. In addition, we also designed the structure of the grating coupler to effectively radiate 60% of the EL spectrum (corresponding to the full width at half maximum of the MoTe_2_ emission) into free space. Crucially, the ability to electrically shift the emission region relaxes alignment precision from the nanometer scale to the micrometer scale (*40*, *47*, *48*). Furthermore, the unique architecture enables light modulation by switching the coupling position of the emission region with the waveguide rather than relying on conventional carrier density modulation. This approach offers the potential to extend the modulation bandwidths beyond the gigahertz regime (see sections S1 and S2 for a detailed explanation). These combined attributes provide a transformative advantage for scalable photonic integration, rendering the device an ideal light source candidate for both on-chip and interchip data transmission.

## DISCUSSION

In this work, we demonstrate a multifunctional MoTe_2_ LET that seamlessly integrates ambipolar switching, dynamically tunable light-emitting, and silicon photonic compatibility. The all-dry fabrication process yields MoTe_2_ LETs with exceptional electrical performance, including an impressive on/off ratio exceeding 10^5^ and balanced carrier mobilities of 39.1 cm^2^ V^−1^ s^−1^ (electron) and 23.2 cm^2^ V^−1^ s^−1^ (hole). The lateral carrier injection design facilitates the integration of MoTe_2_ LETs with silicon photonics. By precisely adjusting the gate and bias voltages, we establish a dynamic p-i-n junction to enable electrically programmable EL positioning. In addition, the radiative recombination region is strategically positioned away from the electrodes, minimizing loss and optimizing performance. This approach allows for the continuous translation of the EL emission along the entire channel length of 15 μm. Our results are expected to benefit the development of the next generation of efficient, electrically pumped, on-chip light sources and lasers. Furthermore, we experimentally verified the integration of MoTe_2_ LETs with a silicon waveguide using the waveguide itself as the G. This architecture achieves waveguide-integrated ambipolar switching and light emission capabilities, offering dual electrical and optical outputs. Spatial programming of the EL position provides a reference method to engineer the coupling between emission and nanostructures. The dynamically reconfigurable light source proposed herein offers broad potential for diverse photonic applications. For instance, electrical modulation of the output optical power enables the implementation of an optical attenuator or modulator by controlling the coupling efficiency to the waveguide. Furthermore, dynamic optical switching or routing can be implemented through precise control of the emission position when integrated with multiple waveguides. Notably, recent progress in the wafer-scale growth of highly crystalline MoTe_2_ on silicon (*49*–*51*), alongside the exploration of silicon-based modulators (*52*) and photodetectors (*53*–*55*), has demonstrated superior performance. Our work fills the critical gap of a light source in all-MoTe_2_ silicon-based photonic integrated circuits, providing a vital pathway toward the industrialization and commercialization of compact on-chip integration of 2D materials.

## MATERIALS AND METHODS

### Device fabrication

Few-layer MoTe_2_ flakes were mechanically exfoliated and positioned onto the surface of polydimethylsiloxane (PDMS). These flakes were then dry transferred onto a 300-nm-thick SiO_2_/Si substrate with a precise alignment system. Then, Au electrodes (with a thickness of 50 nm) were manipulated by PDMS and released accurately onto the MoTe_2_ layer as the D and S electrodes. The *h*-BN flakes were transferred to the top of the channel to provide protection from the environment. Last, the device was annealed in an Ar atmosphere with a temperature of 473 K for 1.5 hours. The preparation process of waveguide-integrated MoTe_2_ LETs was similar to that of the above device. The silicon waveguide and grating couplers were fabricated on a silicon-on-insulator substrate. The patterns were defined by the electron beam lithography on resist and then were etched by inductively coupled plasma. Last, the *h*-BN, MoTe_2_, Au electrodes and *h*-BN were transferred on the waveguide in sequence.

### Electrical and optoelectronic measurements

Electrical and optoelectronic measurements of the fabricated MoTe_2_ LETs were carried out with a semiconductor parameter analyzer (PDA FSpro) at room temperature in ambient conditions. The measurement setup for PL and EL includes a self-built focusing light path and a spectrometer equipped with a thermoelectric-cooled InGaAs 2D photodetector array and a liquid nitrogen–cooled InGaAs 1D photodetector array. The PL of MoTe_2_ was pumped by a continuous wave He-Ne laser at 633 nm. The pumped light was vertically incident onto the sample plane by a 50× objective lens with numerical aperture = 0.42, which also collected the emission light. For the EL spectrum and image measurement, the MoTe_2_ LET was driven by PDA Fspro, and the emission was collected using the measurement setup mentioned above. In the case of photocurrent mapping, the laser beam was focused by the 50× objective lens, and the spot size of the 633 nm is ~2 μm in diameter.

## Supplementary Materials

### This PDF file includes:

Supplementary Text S1 to S3 Figs. S1 to S16 Tables S1 and S2 References
