# Supplementary material for: 2D Material light–emitting transistor with a dynamically controllable emission location for optimized waveguide coupling on silicon
Source: Sci Adv. 2026 Apr 3;12(14):eaeb8783. doi: 10.1126/sciadv.aeb8783 (PMC13048260; doi:10.1126/sciadv.aeb8783)
Supplement: Supplementary file 1 — Supplementary Text S1 to S3 Figs. S1 to S16 Tables S1 and S2 References [file sciadv.aeb8783_sm.pdf]

Science Advances
AAAS

Supplementary Materials for

2D Material light–emitting transistor with a dynamically controllable emission location for optimized waveguide coupling on silicon

Chen Li *et al.*

Corresponding author: Yongzhuo Li, liyongzhuo@tsinghua.edu.cn; Cun-Zheng Ning, ningcunzheng@sztu.edu.cn

Sci. Adv. **12**, eaeb8783 (2026)
DOI: 10.1126/sciadv.aeb8783

This PDF file includes:

Supplementary Text S1 to S3
Figs. S1 to S16
Tables S1 and S2
References

Supplementary Text

Section S1. Estimation of modulation bandwidth of the $MoTe_2$ LET.

The modulation bandwidth of the $MoTe_2$ LET is primarily governed by three factors: the charge carrier transit time (τ_{tr}), the exciton lifetime (τ_e) and the circuit RC time constant (τ_{RC}).
In our proposed $MoTe_2$ LET, the dynamic *p-i-n* junction formed within the channel can significantly reduce τ_{RC}, since the capacitive area in the lateral structure is determined by the extremely small cross-sectional area of the ultrathin $MoTe_2$ layer. The expression for τ_{RC} is given by:

$$\tau_{RC} = R_L \times C \tag{S1}$$

where R_L is the load resistance and C is junction capacitance of *p-i-n* junction, calculated as:

$$C = \frac{\varepsilon_0 \varepsilon_r h W}{L} \tag{S2}$$

Here, ε_0 is the vacuum dielectric constant (8.85×10^{-12} F/m), ε_r is the in-plane dielectric constant, h is the thickness of $MoTe_2$ layer, W and L denote the width and length of channel, respectively.
For the device in Fig. 1B, C is calculated to be approximately 3.22×10^{-6} pF, while for the device in Fig. 4B, C is about 4.27×10^{-6} pF. The resulting τ_{RC} values are extremely small, confirming that τ_{RC} is no longer a limitation factor for the modulation bandwidth of our device.
The τ_{tr} can be calculated according to the following equation:

$$\tau_{tr} = \frac{d}{2\upsilon} = \frac{d^2}{2\mu V} \tag{S3}$$

However, the electric potential within the channel of $MoTe_2$ LET is nonuniform during the operation, meaning that V is a function of the position x along the channel. By applying equation (S2) in the main text, we obtain $V_{eff}(x)$, and subsequently derive $V_{ch}(x)$ using the following relation:

$$V_{ch}(x) = V_g - V_{eff}(x) \tag{S4}$$

Moreover, owing to the difference in electron and hole mobilities and the variation in drift distances for electrons and holes when the recombination region is located at different positions, τ_{tr} is effectively determined by the slower of the two types of carriers injected from both ends. Assuming the ambipolar injection regime described in the main text, where electrons are injected from the S terminal ($x = 0$) and holes from the D terminal ($x = L$), the τ_{tr} when the recombination region is at position x is given by:

$$\tau_{tr} = \max\left(\left| \int_0^x \frac{dx}{\mu_e \times \frac{dV_{ch}}{dx}} \right|, \left| \int_L^{L-x} \frac{dx}{\mu_h \times \frac{dV_{ch}}{dx}} \right| \right) \tag{S5}$$

When the recombination region is located at the center of the channel, corresponding to the $V_{eff}(x)$ illustrated in Fig. 3B. Substituting equation (S4) into equation (S5) yields $\tau_{tr\text{-}e} = 1.56$ ns, $\tau_{tr\text{-}h} = 2.33$ ns. This is equivalent to a resulting transit time of $\tau_{tr} = \max(\tau_{tr\text{-}e}, \tau_{tr\text{-}h}) = 2.33$ ns. The modulation bandwidth is calculated to be 68.32 MHz according to the following equation:

$$f_{3dB\text{-}tr} = \frac{1}{2\pi\tau_{tr}} \tag{S6}$$

The τ_e is estimated around 80 ps according to literature (*56*), which corresponds to 3.9 GHz. Consequently, the current modulation bandwidth is primarily limited by the long channel length of our device. We can achieve a higher modulation bandwidth by further scaling down the channel length and optimizing the position of radiative recombination. As shown in fig. S12, we have systematically calculated the device bandwidth under various recombination regions. Notably, the optimal bandwidth of 84.5 MHz can be achieved when both electrons and holes exhibit equal transit times. Furthermore, for a realistically scaled 1 μm channel length in future waveguide-integrated configurations, our device is theoretically capable of achieving modulation speeds exceeding 1 GHz.

Section S2. Estimation of spatial-switching bandwidth of the recombination region.

The spatial-switching speed of recombination region is different from the modulation bandwidth of the device. This process does not rely on carrier injection from both terminals, but rather on the near-instantaneous local movement of carriers within the recombination zone. This mechanism can significantly enhance the modulation bandwidth from another perspective. Due to the nonuniform distribution of the V_{ch}, when carriers move from position x_1 to x_2, the driving potential difference for electrons and holes are respectively:

$$\Delta V(x) = V_{\mathrm{ch}x_2}(x) - V_{\mathrm{ch}x_1}(x) \tag{S7}$$

Here, the movement speeds of electrons and holes can be expressed as follows:

$$\begin{cases} v_{\mathrm{e}}(x) = \mu_{\mathrm{e}} \left| \dfrac{\mathrm{d}\Delta V(x)}{\mathrm{d}x} \right| \\ v_{\mathrm{h}}(x) = \mu_{\mathrm{h}} \left| \dfrac{\mathrm{d}\Delta V(x)}{\mathrm{d}x} \right| \end{cases} \tag{S8}$$

By referring to equations (S5) and (S6) in section S1, we can calculate the spatial-switching bandwidth of the recombination region. Taking the case where the recombination region is located at the center of the channel as an example, both the average potential difference and the bandwidth within the channel vary with the position of the recombination region, as shown in fig. S13. Within the ±240 nm range around channel center, the switching bandwidth exceeds 7.4 GHz. Under these conditions, the operational limit of the device is ultimately determined by the exciton lifetime.

Section S3. Coupling efficiency of the waveguide-integrated $MoTe_2$ LET.

We utilize the schematic diagram shown in fig. S14 to analyze the entire process and extract the coupling efficiency between the device and the waveguide. With the assistance of the 3D finite-difference time-domain calculations, the entire process can be analyzed into three parts:

1) The section of light source. An *h*-BN/$MoTe_2$/*h*-BN heterostructure is sequentially stacked on top of the waveguide, with an in-plane electric dipole placed within the $MoTe_2$ layer to simulate EL emission. We assume the $I_{\mathrm{all}}(\lambda)$ is the entire intensity of EL emission from the region above the waveguide. A fraction $\xi_{\mathrm{up}}(\lambda)$ of this radiation is emitted upward and collected by an objective lens with a numerical aperture (NA) of 0.42, which has a far-field radiation collection efficiency of $\xi_{\mathrm{up\text{-}NA}}(\lambda)$. The fig. S14B shows the $\xi_{\mathrm{up}}(\lambda)$ and the $\xi_{\mathrm{up\text{-}NA}}(\lambda)$ obtained through the simulation. Due to the presence of the silicon waveguide below the electric dipole, the $\xi_{\mathrm{up}}(\lambda)$

of the emission compared to that in free space is reduced. The overall EL intensity above the waveguide, denoted I_{WG} which represents the integrated EL intensity across the entire wavelength and area of waveguide can be extracted from Fig. 4D. By differentiating the profile of EL spectrum in Fig. 5B, we obtain the spectral intensity $I_{WG}(\lambda)$. Therefore, the total EL intensity emitted by the device in the region above the waveguide is given by:

$$I_{\text{all}}(\lambda) = \frac{I_{\text{WG}}(\lambda)}{\xi_{\text{up}}(\lambda) \times \xi_{\text{up-NA}}(\lambda)} \tag{S9}$$

2) The section of waveguide. The emitter radiates downward and couples into the waveguide with a coupling efficiency of $\xi_{\text{LET-WG}}(\lambda)$, forming a steadily propagating fundamental mode that travels uniformly along both directions. The coupling efficiency of $\xi_{\text{LET-WG}}(\lambda)$ is defined as the ratio of the intensity of the stable transmission mode along the waveguide to the total intensity of the light emission within the waveguide region. As shown in fig. S15A, a 250 μm-long taper is designed to linearly transition in width from 520 nm to 15 μm. The Simulation result confirms that the transmission efficiency $\xi_{\text{WG}}(\lambda)$ exceeds 91%, as shown in fig. S15C. The EL intensity that reaches the grating couplers after transmission $I_{\text{WG-grating}}(\lambda)$ can be calculated as follows:

$$I_{\text{WG-grating}}(\lambda) = I_{\text{all}}(\lambda) \times \xi_{\text{LET-WG}}(\lambda) \times \xi_{\text{WG}}(\lambda) \tag{S10}$$

3) The section of grating coupler. After being adiabatically transformed by the taper, the waveguide mode is diffracted into free space via the grating coupler. We simulate both the transmittance above the grating $\xi_{\text{LG}}(\lambda)$ and its far-field collection efficiency $\xi_{\text{LG-NA}}(\lambda)$ under the actual device parameters (see SEM images and parameters in fig. S10D). Owing to the excellent directionality of the grating coupler, a high collection efficiency can be achieved across a broad spectral range of the emitter, as shown in fig. S14D. The EL intensities of I_{LG} and I_{RG} can be obtained from Fig. 4D. Following the same procedure used for I_{WG} mentioned above, we derive the EL spectral intensities $I_{\text{LG}}(\lambda)$ and $I_{\text{RG}}(\lambda)$. However, due to fabrication-induced asymmetry between the two grating couplers (see in Fig. 5B), we recalibrate the $\xi_{\text{LG}}(\lambda)$ to $\xi_{\text{RG}}(\lambda)$ and $\xi_{\text{LG-NA}}(\lambda)$ to $\xi_{\text{RG-NA}}(\lambda)$ for the right-side grating coupler using the distribution obtained from the better-performing left-side grating coupler. The calibration essentially treats the left-side grating coupler as the ideal reference to correct for losses on the right-side. Since this grating coupler's output is independent of the emitter-to-waveguide coupling process, without such correction, the actual in-coupling intensity of the emitter into the waveguide would be underestimated, leading to reduced calculated coupling efficiency. This allows us to determine the stabilized EL intensity of the propagating mode within the waveguide, denoted as $I_{\text{LET-WG}}(\lambda)$ using the following expressions:

$$I_{\text{LET-WG}}(\lambda) = \frac{I_{\text{WG-grating}}(\lambda)}{\xi_{\text{WG}}(\lambda)} \tag{S11}$$

$$I_{\text{WG-grating}}(\lambda) = \frac{I_{\text{LG}}(\lambda)}{\xi_{\text{LG}}(\lambda) \times \xi_{\text{LG-NA}}(\lambda)} + \frac{I_{\text{RG}}(\lambda)}{\xi_{\text{RG}}(\lambda) \times \xi_{\text{RG-NA}}(\lambda)} = \frac{2 \times I_{\text{LG}}(\lambda)}{\xi_{\text{LG}}(\lambda) \times \xi_{\text{LG-NA}}(\lambda)} \tag{S12}$$

Subsequently, we can calculate the coupling efficiency $\xi_{\text{LET-WG}}(\lambda)$ using the following equation:

$$\xi_{\text{LET-WG}}(\lambda) = \frac{I_{\text{LET-WG}}(\lambda)}{I_{\text{all}}(\lambda)} \tag{S13}$$

As shown in fig. S16a, the $MoTe_2$ LET exhibits a peak coupling efficiency of 21.2% at 1329 nm. Beyond the 1150–1350 nm wavelength range, the intrinsically low EL intensity leads to increased computational uncertainties and significant fluctuations, making the data in these regions less

reliable. Following the formal definition of coupling efficiency, we have replotted the data from Fig. 5A in fig. S16B. Although differences in the definitions of the parameters η and ξ lead to slight shifts in the exact location of the extremum, the overall variation trend remains consistent.

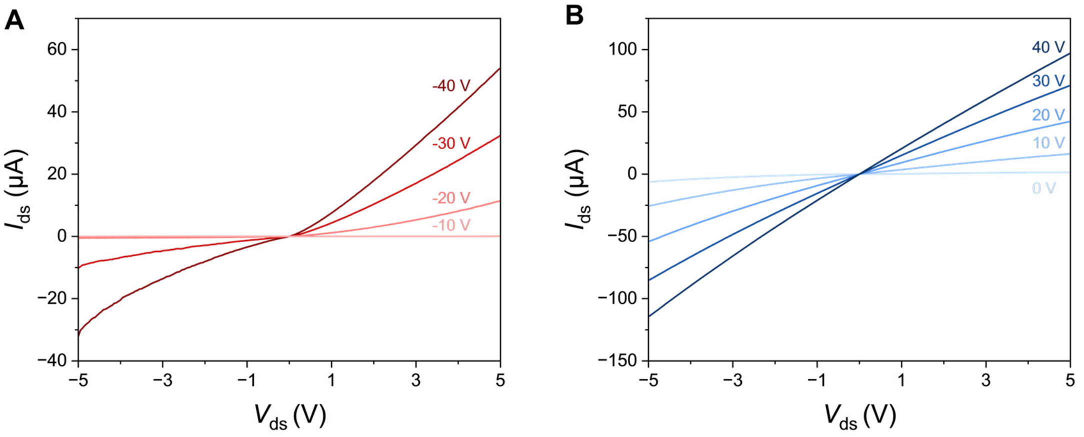

Fig. S1. Output curves at different gate voltages. (**A**) I_{ds}–V_{ds} curves for V_g = –40 V to –10 V. (**B**) I_{ds}–V_{ds} curves for V_g = 0 V to 40 V.

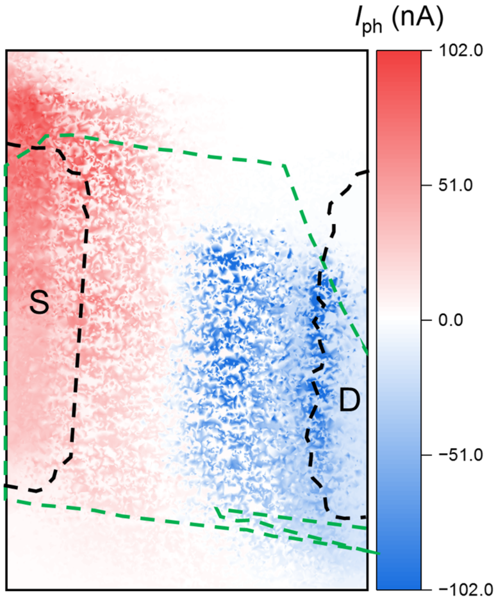

Fig. S2. The photocurrent mapping image under 633 nm light illumination without bias and gate voltages.

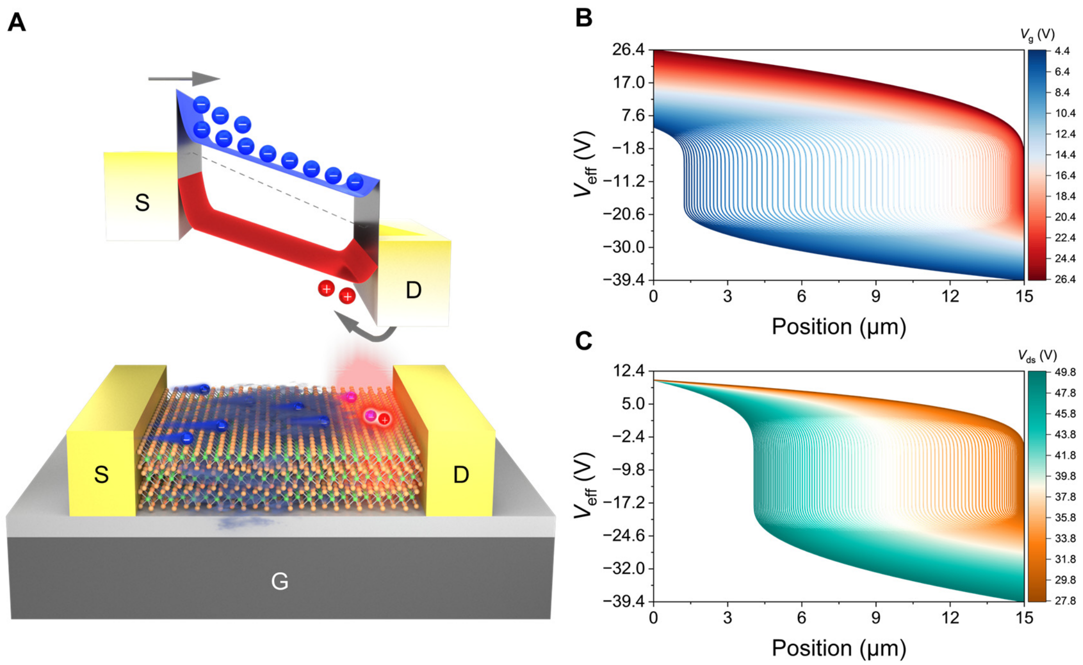

Fig. S3. The principle of uniporlar injection and the distribution relationship of V_{eff}, V_g, and V_{ds} in ambipolar and unipolar injection. (**A**) Schematic diagram of band structure (upper) and injection type (lower) for unipolar injection. The Fermi level of the entire channel is represented by the dashed line. (**B**) The distribution relationship of V_{eff} under different V_g with V_{ds} of 43.8 V. The abrupt V_{eff} transition moves from the S to the D as V_g increases and locates at the middle of the channel under V_g of 11.4 V. (**C**) The distribution relationship of V_{eff} under different V_{ds} with V_g of 10.4 V. The abrupt V_{eff} transition moves from the S to the D as V_{ds} decreases and locates at the middle of the channel under V_{ds} of 41.8 V.

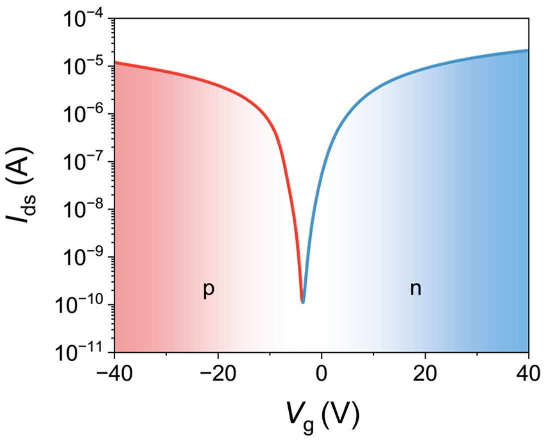

Fig. S4. Transfer curve at V_{ds} = 1.0 V after a period of characterization.

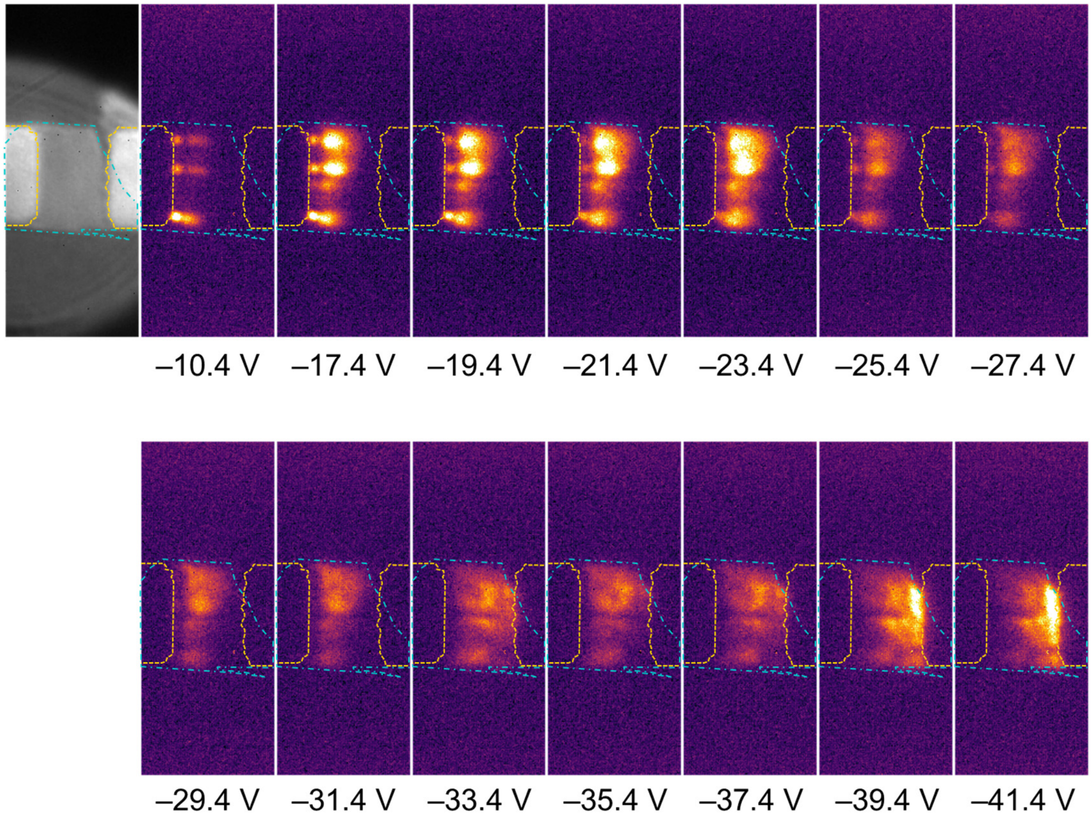

Fig. S5. CCD images (false color) of device and EL emission at fixed V_{ds} = –43.8 V and different V_g. The emission region moves from the S (left) to the D (right) with increasing V_g.

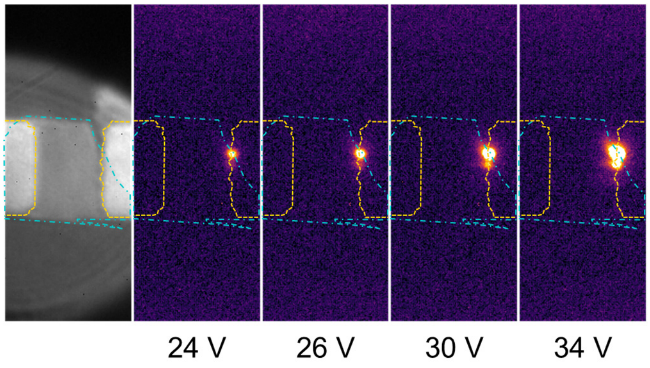

Fig. S6. CCD images (false color) of device and EL emission at unipolar injection with $V_{ds} = V_g$. The emission region is localized at the edge of the electrode.

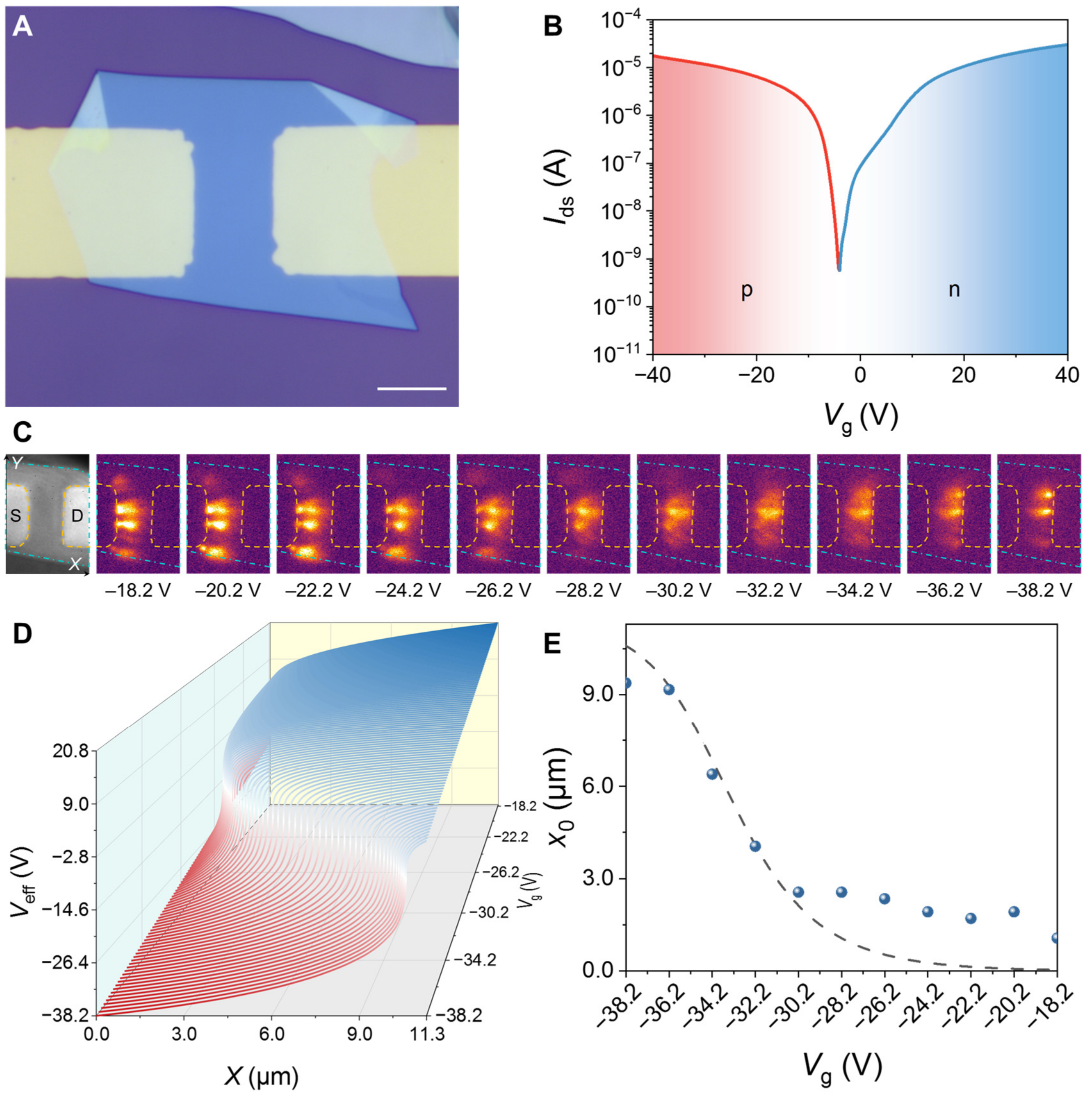

Fig. S7. Electrical and EL properties of device 2. (**A**) Optical microscope image of the device. The scale bar is 10 μm. (**B**) Transfer curve at V_{ds} = 1.0 V. (**C**) CCD images (false color) of device and EL emission at fixed V_{ds} = –39 V and different V_g. The emission region moves from the S (left) to the D (right) with increasing V_g. (**D**) Distribution of V_{eff} under different V_g with V_{ds} of –39 V in ambipolar injection regime. (**E**) EL positions along the channel at different V_g (The blue points are extracted from (A), and the dashed line is extracted from (D)).

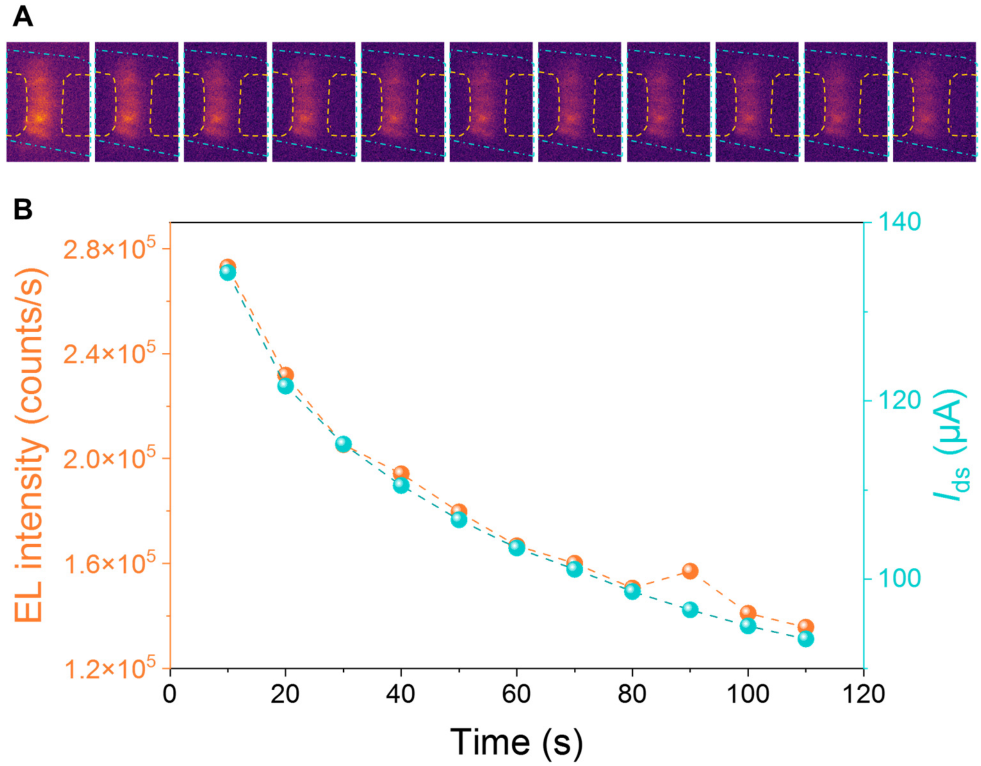

Fig. S8. The operational stability of device 2. (**A**) The continuous emission of EL at fixed V_{ds} = –32 V and V_g = –14.6 V. (**B**) EL intensity (orange) and I_{ds} (cyan) as a function of continuous operation time.

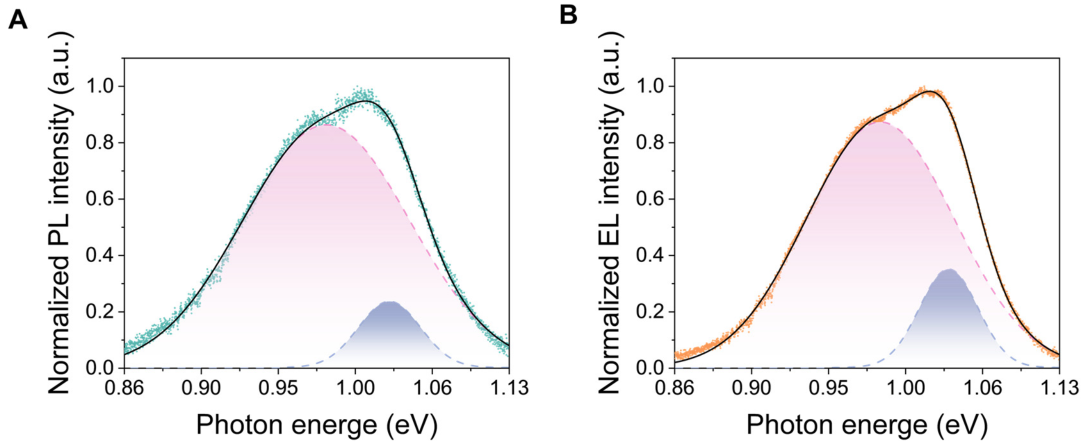

Fig. S9. The fitting results of the normalized emission spectra. The dots represent the experimental data of (**A**) PL and (**B**) EL spectra. The spectra are fitted by two Gaussian curves.

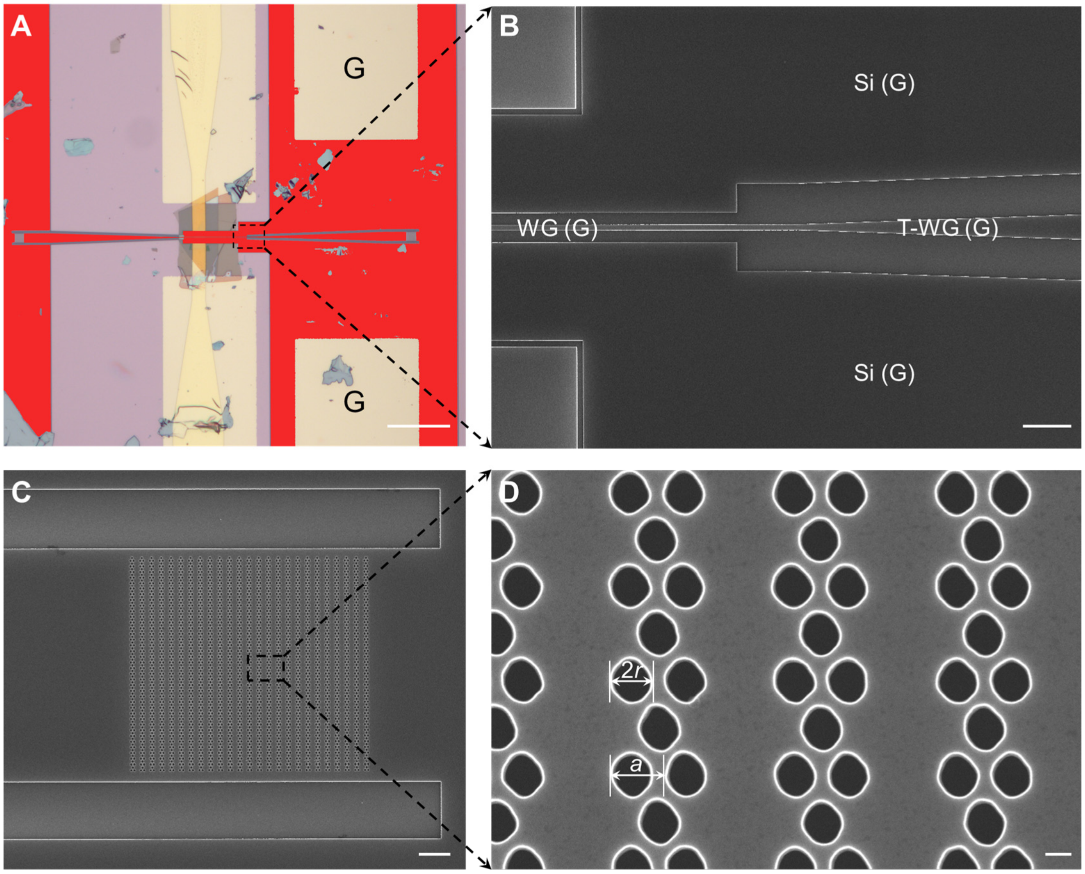

Fig. S10. Optical microscope image of the device architecture and SEM image of the grating coupler. (**A**) Optical microscope image of the waveguide-integrated $MoTe_2$ LET. The red area represents the portion that reaches the same electric potential after applying a V_g to G electrode. The scale bar is 100 μm. (**B**) The SEM image of the straight waveguide and tapered waveguide. The scale bar is 2 μm. (**C**) The SEM image of the grating coupler. The scale bar is 2 μm. (**D**) Zoomed in SEM image of box region in (C). The photonic crystal has a lattice constant a of 210 nm and air-hole radius r of 79.25 nm (r/a = 0.377). The scale bar is 100 nm.

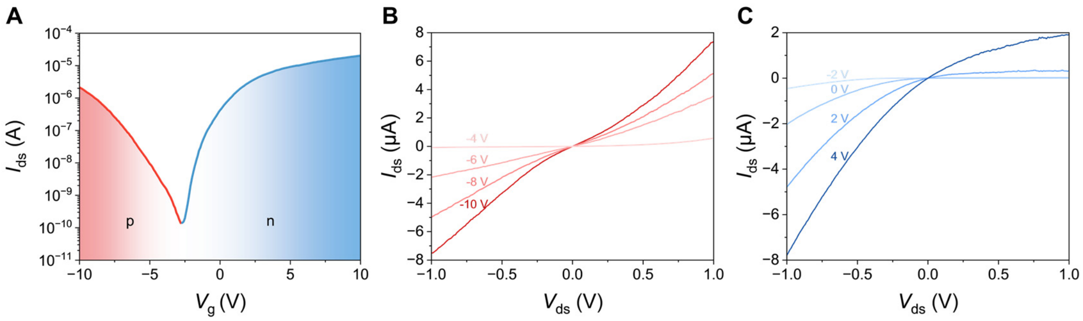

Fig. S11. Electrical characteristics of waveguide-integrated $MoTe_2$ LET. (**A**) Transfer curve at V_{ds} = 1 V. (**B**, **C**) Output curves at different gate voltages.

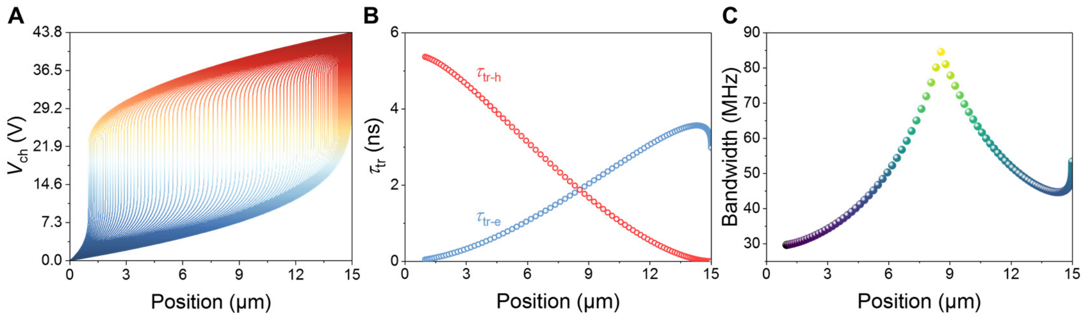

Fig. S12. Estimation of the modulation bandwidth of the $MoTe_2$ LET. (**A**) Distribution of V_{ch} under different V_g with V_{ds} of 43.8 V in ambipolar injection regime. (**B**) The τ_{tr} for electron and hole to drift from the S and D terminal to the recombination region as a function of its position along the channel, respectively. (**C**) The modulation bandwidth as a function of recombination region.

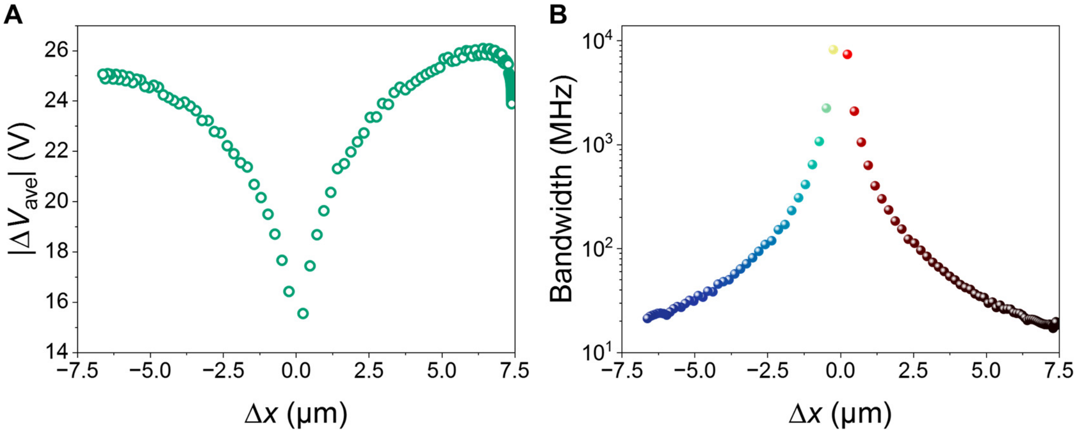

Fig. S13. Estimation of spatial-switching bandwidth of the recombination region. (**A**, **B**) The average potential difference (A) and bandwidth (B) as functions of the switching of the recombination region.

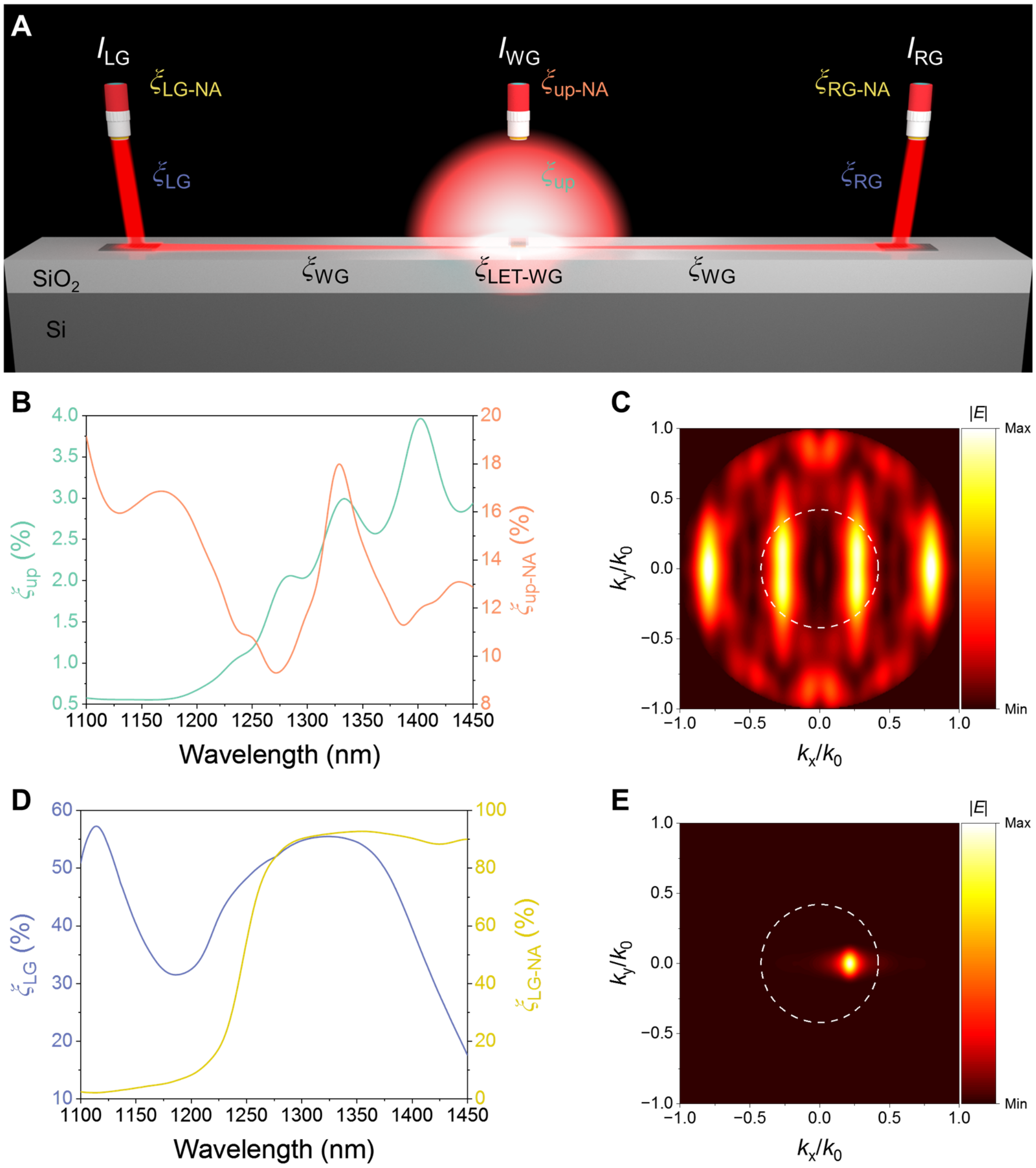

Fig. S14. Coupling efficiency of the waveguide-integrated $MoTe_2$ LET. (**A**) The schematic diagram of the emission from $MoTe_2$ LET coupled with silicon waveguide, combined with simulation and experimental analysis. (**B**) Simulated ξ_{up} and $\xi_{up\text{-}NA}$ of in-plane dipole inside $MoTe_2$ layer located between the two *h*-BN layers above the waveguide. (**C**) Far-field intensity profile of dipole at 1329 nm. (**D**) Simulated ξ_{LG} and $\xi_{LG\text{-}NA}$ of left-side grating coupler. (**E**) Far-field intensity profile of grating coupler at 1329 nm.

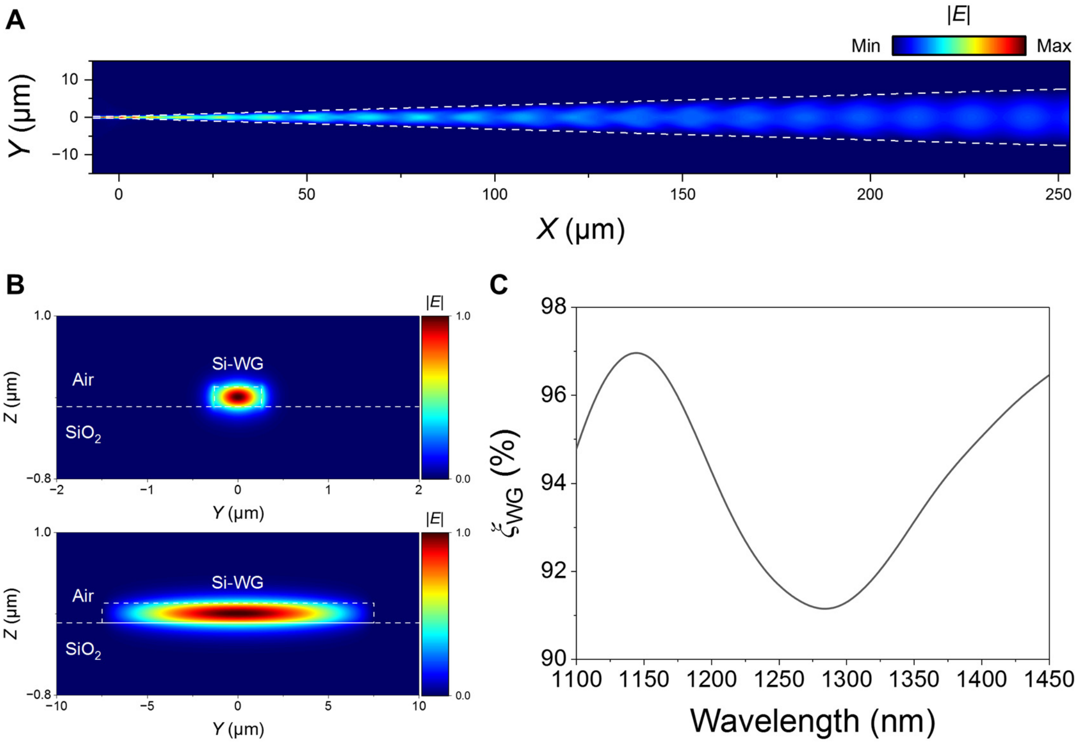

Fig. S15. The simulation results about the tapered waveguide. (**A**) The electric field distribution of the linear tapered waveguide with length of 250 μm. (**B**) The fundamental mode profiles of waveguides with width of 520 nm (upper) and 15 μm (lower), respectively. (**C**) The transmission efficiency ξ_{WG} (λ) of the linear tapered waveguide.

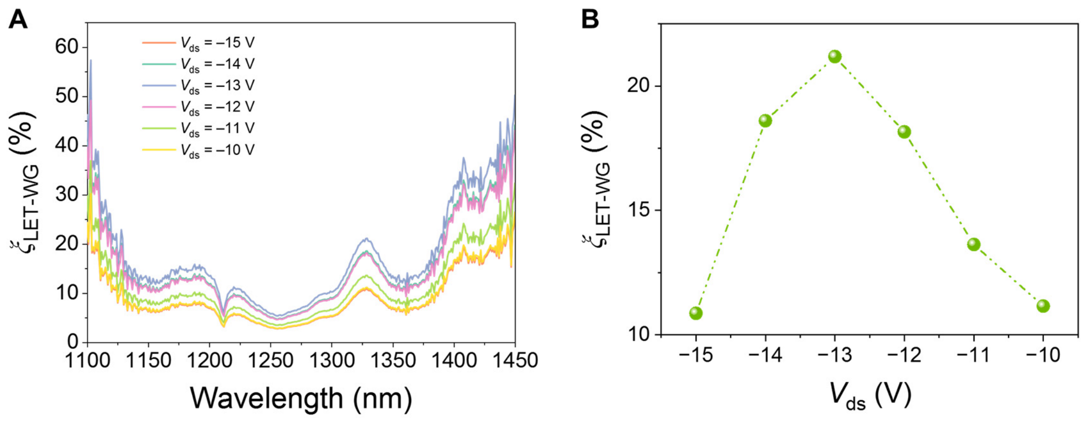

Fig. S16. The EL controllable characteristics of the waveguide-integrated $MoTe_2$ LET in the main text. (**A**) The coupling efficiency $\xi_{\text{LET-WG}}$ (λ) at fixed $V_g = -12$ V and different V_{ds}. (**B**) The variation of the maximum coupling efficiency $\xi_{\text{LET-WG}}$ (1329 nm) at fixed $V_g = -12$ V and different V_{ds}.

Table S1. Electrical and EL characteristics of $MoTe_2$ LETs.

Device	Dielectric	On/off ratio (p/n)	V_{th} (p/n, V)	Mobility (p/n, $cm^2 V^{-1} s^{-1}$)	V_{ds}/V_g[§] (V)
#1[*]	SiO_2	$5.54\times10^5/1.64\times10^6$	–17.2 V/–6.8 V	23.2/39.1	43.8/11.6
#2[†]	SiO_2	$3.16\times10^4/5.36\times10^4$	–4.0 V/–3.6 V	33.8/50.9	–39/–33.4
#3[‡]	*h*-BN	$1.53\times10^4/1.45\times10^5$	–2.5 V/–2.1 V	7.2/15.5	–14/–12

[*] The optical microscopic image of the device is shown in Fig. 1B.
[†] The optical microscopic image of the device is shown in fig. S7A.
[‡] The optical microscopic image of the device is shown in Fig. 4B.
[§] This parameter represents V_{ds} and V_g when the emission region is located in the middle of the channel.

Table S2. Comparisons of figures of merit for 2D materials LEDs and their waveguide-integrated structure.

Materials	Emission mechanism	Emission central wavelength (nm)	Waveguide structure	Photonic structure efficiency*
$MoTe_2$ (*40*)	p-n junction	1160	photonic crystal waveguide	5%
BP (*41*)	vdW heterostructures	3640	silicon waveguide	13.8%†
WSe_2 (*42*)	tunnel junction	765	h-BN waveguide	1.23%‡
WSe_2 (*43*)	p-n junction	739	CdS nanoribbon	13.1%
WSe_2 (*44*)	AC-driven	750	Ag nanowire	-
WSe_2 (*45*)	AC-driven	750	MoO_3 waveguide	5.4%§
Graphene (*46*)	tunnel junction	800	Ag nanowire	36%
$MoTe_2$ This work	LET	1300	silicon waveguide	34.7%~67.2%

* The efficiency of photonic sturcture is defined according to the reference (*40*).

† The measured emission intensity from the right (left) grating coupler is 10% (6%) with respect to the central BP emissive region.

‡ The photon number detected at the waveguide end is divided by the photon number at the LED region. The uncorrected total coupling efficiency is 1.25 %, and the corrected total coupling efficiency is determined to be 0.3%.

§ The intensity ratio between edge-scattered EL at the MoO_3 nanoribbon terminus and direct emission from the WSe_2 monolayer on top of the MoO_3 nanoribbon is 5.7 %.

REFERENCES

1. J. Pu, T. Takenobu, Monolayer transition metal dichalcogenides as light sources. *Adv. Mater.* **30**, 1707627 (2018).

2. Z. Cheng, R. Cao, K. Wei, Y. Yao, X. Liu, J. Kang, J. Dong, Z. Shi, H. Zhang, X. Zhang, 2D Materials enabled next-generation integrated optoelectronics: From fabrication to applications. *Adv. Sci.* **8**, e2003834 (2021).

3. Y. Li, J. Zhang, D. Huang, H. Sun, F. Fan, J. Feng, Z. Wang, C. Z. Ning, Room-temperature continuous-wave lasing from monolayer molybdenum ditelluride integrated with a silicon nanobeam cavity. *Nat. Nanotechnol.* **12**, 987–992 (2017).

4. S. Wu, S. Buckley, J. R. Schaibley, L. Feng, J. Yan, D. G. Mandrus, F. Hatami, W. Yao, J. Vuckovic, A. Majumdar, X. Xu, Monolayer semiconductor nanocavity lasers with ultralow thresholds. *Nature* **520**, 69–72 (2015).

5. Y. Liu, H. Fang, A. Rasmita, Y. Zhou, J. Li, T. Yu, Q. Xiong, N. Zheludev, J. Liu, W. Gao, Room temperature nanocavity laser with interlayer excitons in 2D heterostructures. *Sci. Adv.* **5**, eaav4506 (2019).

6. H. Fang, J. Liu, Q. Lin, R. Su, Y. Wei, T. F. Krauss, J. Li, Y. Wang, X. Wang, Laser-like emission from a sandwiched $MoTe_2$ heterostructure on a silicon single-mode resonator. *Adv. Opt. Mater.* **7**, 1900538 (2019).

7. H. Fang, J. Liu, H. Li, L. Zhou, L. Liu, J. Li, X. Wang, T. F. Krauss, Y. Wang, 1305 nm few-layer $MoTe_2$-on-silicon laser-like emission. *Laser Photon. Rev.* **12**, 1800015 (2018).

8. C. Errando-Herranz, E. Scholl, R. Picard, M. Laini, S. Gyger, A. W. Elshaari, A. Branny, U. Wennberg, S. Barbat, T. Renaud, M. Sartison, M. Brotons-Gisbert, C. Bonato, B. D. Gerardot, V. Zwiller, K. D. Jöns, Resonance fluorescence from waveguide-coupled, strain-localized, two-dimensional quantum emitters. *ACS Photonics* **8**, 1069–1076 (2021).

9. H. Chen, V. Corboliou, A. S. Solntsev, D. Y. Choi, M. A. Vincenti, D. de Ceglia, C. de Angelis, Y. Lu, D. N. Neshev, Enhanced second-harmonic generation from two-dimensional $MoSe_2$ on a silicon waveguide. *Light Sci. Appl.* **6**, e17060 (2017).

10. P. Tonndorf, O. Del Pozo-Zamudio, N. Gruhler, J. Kern, R. Schmidt, A. I. Dmitriev, A. P. Bakhtinov, A. I. Tartakovskii, W. Pernice, S. Michaelis de Vasconcellos, R. Bratschitsch, On-chip waveguide coupling of a layered semiconductor single-photon source. *Nano Lett.* **17**, 5446–5451 (2017).

11. Y. Ye, Z. J. Wong, X. Lu, X. Ni, H. Zhu, X. Chen, Y. Wang, X. Zhang, Monolayer excitonic laser. *Nat. Photonics* **9**, 733–737 (2015).

12. O. Salehzadeh, M. Djavid, N. H. Tran, I. Shih, Z. Mi, Optically pumped two-dimensional MoS_2 lasers operating at room-temperature. *Nano Lett.* **15**, 5302–5306 (2015).

13. J. C. Reed, A. Y. Zhu, H. Zhu, F. Yi, E. Cubukcu, Wavelength tunable microdisk cavity light source with a chemically enhanced MoS_2 emitter. *Nano Lett.* **15**, 1967–1971 (2015).

14. J. S. Ross, P. Klement, A. M. Jones, N. J. Ghimire, J. Yan, D. G. Mandrus, T. Taniguchi, K. Watanabe, K. Kitamura, W. Yao, D. H. Cobden, X. Xu, Electrically tunable excitonic light-emitting diodes based on monolayer WSe_2 p-n junctions. *Nat. Nanotechnol.* **9**, 268–272 (2014).

15. A. Pospischil, M. M. Furchi, T. Mueller, Solar-energy conversion and light emission in an atomic monolayer p-n diode. *Nat. Nanotechnol.* **9**, 257–261 (2014).

16. B. W. Baugher, H. O. Churchill, Y. Yang, P. Jarillo-Herrero, Optoelectronic devices based on electrically tunable p-n diodes in a monolayer dichalcogenide. *Nat. Nanotechnol.* **9**, 262–267 (2014).

17. Y. J. Zhang, T. Oka, R. Suzuki, J. T. Ye, Y. Iwasa, Electrically switchable chiral light-emitting transistor. *Science* **344**, 725–728 (2014).

18. S. Jo, N. Ubrig, H. Berger, A. B. Kuzmenko, A. F. Morpurgo, Mono- and bilayer WS_2 light-emitting transistors. *Nano Lett.* **14**, 2019–2025 (2014).

19. J. Feng, Y. Li, J. Zhang, Y. Tang, H. Sun, L. Gan, C. Z. Ning, Injection-free multiwavelength electroluminescence devices based on monolayer semiconductors driven by an alternating field. *Sci. Adv.* **8**, eabl5134 (2022).

20. Y. Liu, J. Guo, E. Zhu, L. Liao, S. J. Lee, M. Ding, I. Shakir, V. Gambin, Y. Huang, X. Duan, Approaching the Schottky-Mott limit in van der Waals metal-semiconductor junctions. *Nature* **557**, 696–700 (2018).

21. D. Qu, X. Liu, M. Huang, C. Lee, F. Ahmed, H. Kim, R. S. Ruoff, J. Hone, W. J. Yoo, Carrier-Type Modulation and Mobility Improvement of Thin $MoTe_2$. *Adv. Mater.* **29**, 1606433 (2017).

22. H. Xu, S. Fathipour, E. W. Kinder, A. C. Seabaugh, S. K. Fullerton-Shirey, Reconfigurable ion gating of 2H-$MoTe_2$ field-effect transistors using poly(ethylene oxide)-$CsClO_4$ solid polymer electrolyte. *ACS Nano* **9**, 4900–4910 (2015).

23. C. Ruppert, B. Aslan, T. F. Heinz, Optical properties and band gap of single- and few-layer $MoTe_2$ crystals. *Nano Lett.* **14**, 6231–6236 (2014).

24. L. Yin, X. Zhan, K. Xu, F. Wang, Z. Wang, Y. Huang, Q. Wang, C. Jiang, J. He, Ultrahigh sensitive $MoTe_2$ phototransistors driven by carrier tunneling. *Appl. Phys. Lett.* **108**, 043503 (2016).

25. Z. Yin, H. Li, H. Li, L. Jiang, Y. Shi, Y. Sun, G. Lu, Q. Zhang, X. Chen, H. Zhang, Single-layer MoS_2 phototransistors. *ACS Nano* **6**, 74–80 (2012).

26. Y. F. Lin, Y. Xu, S. T. Wang, S. L. Li, M. Yamamoto, A. Aparecido-Ferreira, W. Li, H. Sun, S. Nakaharai, W. B. Jian, K. Ueno, K. Tsukagoshi, Ambipolar $MoTe_2$ transistors and their applications in logic circuits. *Adv. Mater.* **26**, 3263–3269 (2014).

27. H. Huang, J. Wang, W. Hu, L. Liao, P. Wang, X. Wang, F. Gong, Y. Chen, G. Wu, W. Luo, H. Shen, T. Lin, J. Sun, X. Meng, X. Chen, J. Chu, Highly sensitive visible to infrared $MoTe_2$ photodetectors enhanced by the photogating effect. *Nanotechnology* **27**, 445201 (2016).

28. S. Larentis, B. Fallahazad, H. C. P. Movva, K. Kim, A. Rai, T. Taniguchi, K. Watanabe, S. K. Banerjee, E. Tutuc, Reconfigurable complementary monolayer $MoTe_2$ field-effect transistors for integrated circuits. *ACS Nano* **11**, 4832–4839 (2017).

29. A. Ortiz-Conde, F. J. García Sánchez, J. J. Liou, A. Cerdeira, M. Estrada, Y. Yue, A review of recent MOSFET threshold voltage extraction methods. *Microelectron. Reliab.* **42**, 583–596 (2002).

30. R. Peng, Y. Wu, B. Wang, R. Shi, L. Xu, T. Pan, J. Guo, B. Zhao, C. Song, Z. Fan, C. Wang, P. Zhou, S. Fan, K. Liu, Programmable graded doping for reconfigurable molybdenum ditelluride devices. *Nat. Electron.* **6**, 852–861 (2023).

31. Z. Qin, H. Gao, H. Dong, W. Hu, Organic light-emitting transistors entering a new development stage. *Adv. Mater.* **33**, e2007149 (2021).

32. J. Kim, K. Cho, J. Pak, W. Lee, J. Seo, J. K. Kim, J. Shin, J. Jang, K. Y. Baek, J. Lee, S. Chung, K. Kang, T. Lee, Channel-length-modulated avalanche multiplication in ambipolar WSe_2 field-effect transistors. *ACS Nano* **16**, 5376–5383 (2022).

33. J. Zaumseil, R. H. Friend, H. Sirringhaus, Spatial control of the recombination zone in an ambipolar light-emitting organic transistor. *Nat. Mater.* **5**, 69–74 (2005).

34. T. Takahashi, T. Takenobu, J. Takeya, Y. Iwasa, Ambipolar light-emitting transistors of a tetracene single crystal. *Adv. Funct. Mater.* **17**, 1623–1628 (2007).

35. Y. J. Zhang, J. T. Ye, Y. Yomogida, T. Takenobu, Y. Iwasa, Formation of a stable *p-n* junction in a liquid-gated MoS_2 ambipolar transistor. *Nano Lett.* **13**, 3023–3028 (2013).

36. E. C. P. Smits, T. D. Anthopoulos, S. Setayesh, E. van Veenendaal, R. Coehoorn, P. W. M. Blom, B. de Boer, D. M. de Leeuw, Ambipolar charge transport in organic field-effect transistors. *Phys. Rev. B* **73**, 205316 (2006).

37. N. Higashitarumizu, S. Tajima, J. Kim, M. Cai, A. Javey, Long operating lifetime mid-infrared LEDs based on black phosphorus. *Nat. Commun.* **14**, 4845 (2023).

38. Y. J. Sun, D. Wang, Z. G. Shuai, Indirect-to-direct band gap crossover in few-layer transition metal dichalcogenides: A theoretical prediction. *J. Phys. Chem. C* **120**, 21866–21870 (2016).

39. L. Liu, M. Pu, K. Yvind, J. M. Hvam, High-efficiency, large-bandwidth silicon-on-insulator grating coupler based on a fully-etched photonic crystal structure. *Appl. Phys. Lett.* **96**, 051126 (2010).

40. Y. Q. Bie, G. Grosso, M. Heuck, M. M. Furchi, Y. Cao, J. Zheng, D. Bunandar, E. Navarro-Moratalla, L. Zhou, D. K. Efetov, T. Taniguchi, K. Watanabe, J. Kong, D. Englund, P. Jarillo-Herrero, A $MoTe_2$-based light-emitting diode and photodetector for silicon photonic integrated circuits. *Nat. Nanotechnol.* **12**, 1124–1129 (2017).

41. T. Y. Chang, Y. Chen, D. I. Luo, J. X. Li, P. L. Chen, S. Lee, Z. Fang, W. Q. Li, Y. Y. Zhang, M. Li, A. Majumdar, C. H. Liu, Black phosphorus mid-infrared light-emitting diodes integrated with silicon photonic waveguides. *Nano Lett.* **20**, 6824–6830 (2020).

42. R. Khelifa, S. Shan, A. J. Moilanen, T. Taniguchi, K. Watanabe, L. Novotny, WSe_2 light-emitting device coupled to an h-BN waveguide. *ACS Photonics* **10**, 1328–1333 (2023).

43. X. Yang, R. Wu, B. Zheng, Z. Luo, W. You, H. Liu, L. Li, Y. Zhang, Q. Tan, D. Liang, Y. Chen, J. Qu, X. Yi, X. Wang, J. Zhou, H. Duan, S. Wang, S. Chen, A. Pan, A waveguide-integrated two-dimensional light-emitting diode based on p-type WSe_2/n-type CdS nanoribbon heterojunction. *ACS Nano* **16**, 4371–4378 (2022).

44. X. Yi, J. Zhai, Z. Luo, H. Liu, Y. Liu, X. Yang, Q. Tan, J. Qu, B. Liu, Q. Jiang, R. Sun, Q. Zhang, Y. Jiang, S. Chen, A. Pan, AC-driven plasmon waveguide integrated electroluminescent device. *Adv. Opt. Mater.* **12**, 2400647 (2024).

45. W. Li, Y. Zhang, G. Zhu, L. Zhang, M. Ge, S. Liu, Y. Hu, T. Wang, W. Du, Strain-enhanced and waveguide-integrated electroluminescence from a 2D monolayer semiconductor. *Adv. Opt. Mater.* **13**, 2500336 (2025).

46. L. Liu, A. V. Krasavin, J. Li, L. Li, L. Yang, X. Guo, D. Dai, A. V. Zayats, L. Tong, P. Wang, Waveguide-integrated light-emitting metal-insulator-graphene tunnel junctions. *Nano Lett.* **23**, 3731–3738 (2023).

47. F. Pyatkov, V. Fütterling, S. Khasminskaya, B. S. Flavel, F. Hennrich, M. M. Kappes, R. Krupke, W. H. P. Pernice, Cavity-enhanced light emission from electrically driven carbon nanotubes. *Nat. Photonics* **10**, 420–427 (2016).

48. S. Khasminskaya, F. Pyatkov, B. S. Flavel, W. H. Pernice, R. Krupke, Waveguide-integrated light-emitting carbon nanotubes. *Adv. Mater.* **26**, 3465–3472 (2014).

49. X. Xu, Y. Pan, S. Liu, B. Han, P. Gu, S. Li, W. Xu, Y. Peng, Z. Han, J. Chen, P. Gao, Y. Ye, Seeded 2D epitaxy of large-area single-crystal films of the van der Waals semiconductor 2H $MoTe_2$. *Science* **372**, 195–200 (2021).

50. S. Song, A. Yoon, S. Jang, J. Lynch, J. Yang, J. Han, M. Choe, Y. H. Jin, C. Y. Chen, Y. Cheon, J. Kwak, C. Jeong, H. Cheong, D. Jariwala, Z. Lee, S. Y. Kwon, Fabrication of *p*-type 2D single-crystalline transistor arrays with Fermi-level-tuned van der Waals semimetal electrodes. *Nat. Commun.* **14**, 4747 (2023).

51. Y. Pan, T. Jian, P. Gu, Y. Song, Q. Wang, B. Han, Y. Ran, Z. Pan, Y. Li, W. Xu, P. Gao, C. Zhang, J. He, X. Xu, Y. Ye, Precise *p*-type and *n*-type doping of two-dimensional semiconductors for monolithic integrated circuits. *Nat. Commun.* **15**, 9631 (2024).

52. H. Cao, H. Chen, Y. Pan, M. Ding, B. Pan, W. Zhao, H. Li, Z. Yu, Y. Ye, D. Dai, Efficient and fast all-optical modulator with in situ grown $MoTe_2$ nanosheets on silicon. *ACS Appl. Nano Mater.* **6**, 838–845 (2023).

53. C. Li, R. Tian, X. Chen, L. Gu, Z. Luo, Q. Zhang, R. Yi, Z. Li, B. Jiang, Y. Liu, A. Castellanos-Gomez, S. J. Chua, X. Wang, Z. Sun, J. Zhao, X. Gan, Waveguide-integrated $MoTe_2$ *p–i–n* homojunction photodetector. *ACS Nano* **16**, 20946–20955 (2022).

54. R. Maiti, C. Patil, M. A. S. R. Saadi, T. Xie, J. G. Azadani, B. Uluutku, R. Amin, A. F. Briggs, M. Miscuglio, D. Van Thourhout, S. D. Solares, T. Low, R. Agarwal, S. R. Bank, V. J. Sorger,

Strain-engineered high-responsivity $MoTe_2$ photodetector for silicon photonic integrated circuits. *Nat. Photonics* **14**, 578–584 (2020).

55. N. Flory, P. Ma, Y. Salamin, A. Emboras, T. Taniguchi, K. Watanabe, J. Leuthold, L. Novotny, Waveguide-integrated van der Waals heterostructure photodetector at telecom wavelengths with high speed and high responsivity. *Nat. Nanotechnol.* **15**, 118–124 (2020).

56. S. Pan, W. Kong, J. Liu, X. Ge, P. Zereshki, S. Hao, D. He, Y. Wang, H. Zhao, Understanding spatiotemporal photocarrier dynamics in monolayer and bulk $MoTe_2$ for optimized optoelectronic devices. *ACS Appl. Nano Mater.* **2**, 459–464 (2018).
